# Highly oxidized products from the atmospheric reaction of hydroxyl radicals with toluene

**DOI:** 10.1038/s42004-026-02230-0

**Published:** 2026-10-08

**Authors:** Torsten Berndt

**Affiliations:** Atmospheric Chemistry Department (ACD), Leibniz Institute for Tropospheric Research (TROPOS), Leipzig, Germany

**Keywords:** Atmospheric chemistry, Reaction kinetics and dynamics

## Abstract

Simple aromatics, largely toluene (C_7_H_8_) and benzene (C_6_H_6_), are emitted into Earth´s atmosphere by different sources. Their degradation initiated by the gas-phase reaction with hydroxyl radicals (OH) forms mainly bicyclic peroxy radicals (BCP) besides phenol-type compounds. The formation of highly oxidized products with at least 6 O atoms (HOMs) starting from BCP is currently controversial. Here, the experimental observation of HOMs and other products is reported for near-atmospheric conditions focusing on OH + toluene. Autoxidation processes starting from BCP generate C_7_H_9_O_7_ and C_7_H_9_O_9_ radicals with molar yields of ${0.25}_{\,-0.13}^{\,+0.25}\, \%$ and ${0.12}_{\,-0.06}^{\,+0.12}\, \%$, respectively. Labeling experiments revealed that the ring H atoms are involved in autoxidation. The atmospherically relevant reaction of C_7_H_9_O_7_ and C_7_H_9_O_9_ with NO almost exclusively forms organic nitrates as HOM products, C_7_H_9_O_6-8_NO_2_, with a total molar yield ≤ 0.1%. Other HOMs, such as C_7_H_8_O_8_ and C_7_H_8_O_10_, represent only small trace products. Results from OH + benzene confirmed the findings from the toluene system pointing to a minor relevance of both aromatics for HOM generation.

## Introduction

Toluene (C_7_H_8_) and benzene (C_6_H_6_) represent the most important emissions of aromatics with a global annual rate of about 8 million metric tons of carbon each arising mainly from anthropogenic sources and biomass burning^1,2^. Their atmospheric degradation proceeds almost exclusively via the gas-phase reaction with hydroxyl (OH) radicals. Here, OH addition to the aromatic ring clearly dominates over H-abstraction on the side chain (if present).

For OH + toluene, the OH addition mainly takes place in *ortho* position with branching ratios *ortho* / *meta* / *para* / *ipso* = 59 - 70% / 5% / 5 - 14% / 7 - 15% based on theoretical calculations^3,4^. The lesser H-abstraction on the side chain amounts to 7 - 12%^3,4^.

**Scheme 1:**
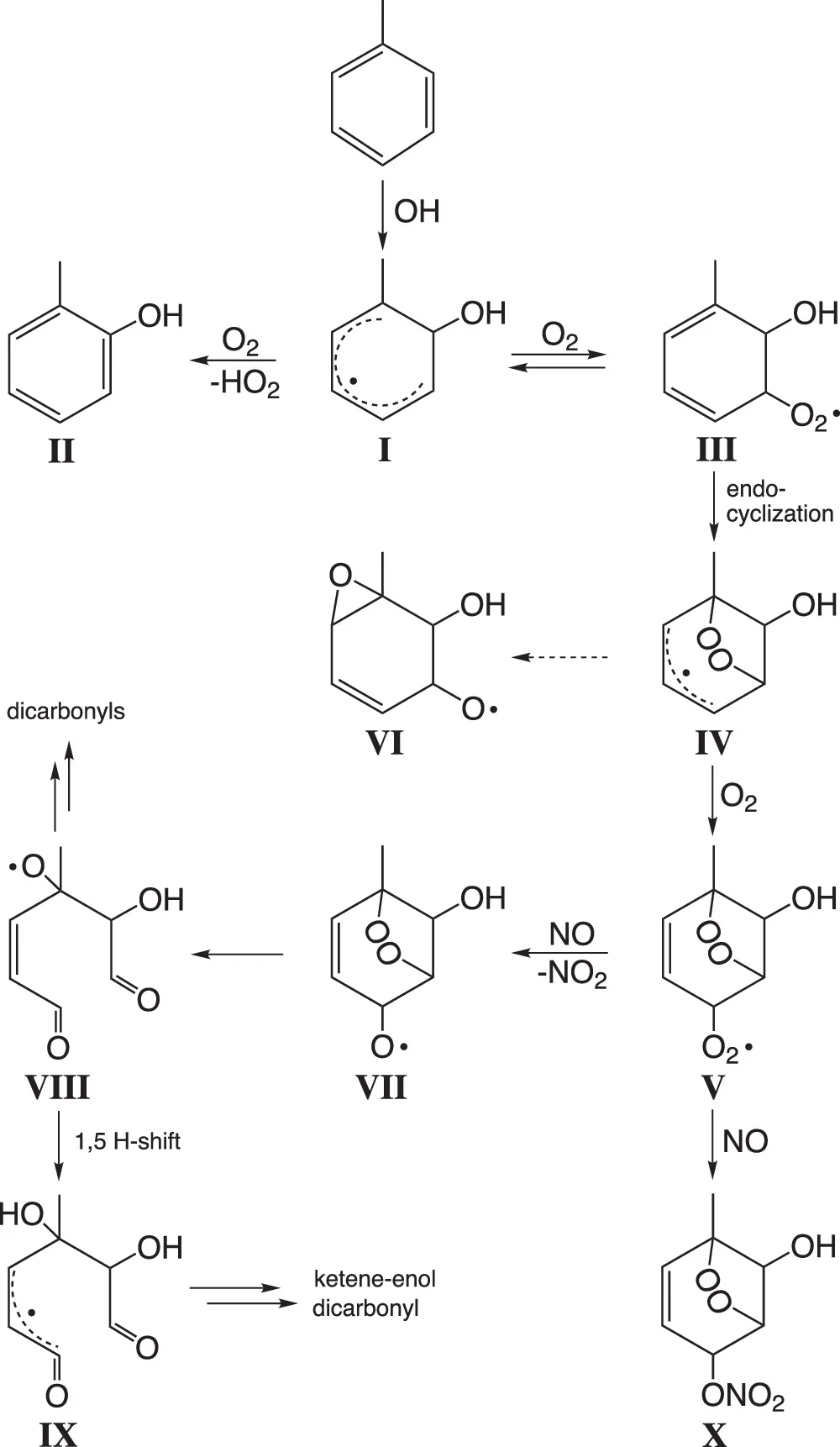
First steps of the OH radical-initiated oxidation of toluene based on literature data. The scheme considers the most abundant OH addition in *ortho* position. H-abstraction from the methyl group is not shown. The dashed reaction arrow indicates a pathway which is likely less important, multiple arrows represent complex reactions to the products.

According to the current knowledge in the literature, OH addition forms an adduct **I** that further reacts with O_2_ in the atmosphere leading either to phenol-type products **II**^5–9^ or reversibly to a RO_2_ radical **III**^10–12^, see Scheme 1 for OH + toluene with OH attack in *ortho* position. The formed RO_2_ radical **III** irreversibly undergoes endo-cyclization forming the allyl species **IV** that further adds O_2_ yielding a bicyclic peroxy radical (BCP) **V**^7,10,12–15^. Formation of an epoxy-alkoxy radical **VI** starting from **IV** was proposed as a competitive route to BCP formation^16^. However, this path is energetically not favored and its relevance is not supported by results of end-product analysis^7,13,15,17^.

The atmospheric fate of BCP **V** is governed by their reaction with NO leading to the corresponding alkoxy radicals **VII** which further reacts via β-scission and breaking of the O-O bridge bond forming the aliphatic alkoxy radical **VIII**. This radical can either further decompose forming finally dicarbonylic products after cleavage of the carbon backbone, i.e., glyoxal, methylglyoxal, 1,4-butenedial or 4-oxo-2-pentenal, or **VIII** undergoes 1,5 H-shift leading to the allylic compound **IX**^7,18^. **IX** further decomposes forming a dicarbonyl along with a ketene-enol species^7,18^ or **IX** adds O_2_. The reported molar yields of dicarbonylic products from different studies are in good agreement^6,7,9,19–22^. On the other hand, the knowledge regarding the corresponding organic nitrate **X** formation from BCP + NO is very sparse and resulting yields are uncertain^7,14,23^. Reported molar yields are 1.2 - 1.6%^14^ and 2.9%^23^ for the toluene reaction and < 0.1%^7^ and 3.4%^23^ in the benzene case.

Despite the big effort in the last decades, the atmospheric degradation process of toluene and benzene is not fully understood up to now. There is still a substantial shortfall in the carbon balance. Reported products so far are able to amount to 70 – 80% of reacted carbon^4–7,9,19–23^.

The degradation scheme (Scheme 1) does not consider possible autoxidation steps, which can form highly oxidized products bearing 6 O atoms or more (HOMs)^24–26^. HOM formation has been experimentally shown to be important in a couple of atmospheric oxidation processes^25^. For the reaction of OH radicals with toluene and benzene, however, the information on HOM formation given in the literature is inconsistent. There are open questions concerning the mechanistic understanding of the autoxidation steps, the importance of different HOM-RO_2_ radicals in this process and the HOM product distribution under dominating RO_2_ + NO conditions relevant in the atmosphere.

In principle, if starting from the BCP **V**, internal H-abstraction as well as endo-cyclization are hindered due to the inflexible bicyclic ring structure. Thus, rapid autoxidation steps cannot be expected.

First, based on theoretical calculations, Wang et al.^27^ proposed H-abstraction from the side chain of BCP of toluene, ethylbenzene etc. with a rate coefficient of 0.026 s^-1^ at 298 K in the case of the toluene-derived BCP formed from OH attack in *para* position. The expected product signal of C_7_H_9_O_7_ from the first BCP autoxidation step was experimentally observed as well as C_7_H_9_O_9_ from a consecutive reaction. Formation yields of HOM-RO_2_ radicals were not determined^27^. Molteni et al.^28^ reported a total molar HOM yield of 0.1% from OH + toluene experiments under HO_2_ radical-rich conditions and 0.2% for OH + benzene. In a chamber study, Garmash et al.^29^ observed molar HOM yields of 4.1 – 14% from OH + benzene under conditions of multiple OH reactions as well as possible ozonolysis reactions of products. Molar HOM yields of about 1 – 2% from OH + toluene were reported from another chamber study conducted under similar complex reaction conditions^30^. In our laboratory, molar HOM yields of ${0.65}_{\,-0.33}^{\,+0.65}\, \%$ (mainly C_7_H_9_O_7_) for OH + toluene and < 0.1% for OH + benzene were measured in flow-tube experiments under low-NO conditions and a reaction time of 7.9 s^31^. More recently, Iyer et al.^32^ proposed a new mechanism of HOM formation for OH + toluene starting from the OH attack in *ipso* position followed by ring opening of the resulting BCP. Rapid formation of C_7_H_9_O_9_ radicals was stated based on theoretical calculations. Experimental support came from low-NO experiments which showed a strong C_7_H_9_O_9_ signals along with traces of C_7_H_9_O_7_^32^. The latter is surprising because C_7_H_9_O_7_ and its products represented a main HOM fraction in the experiments reported so far^27–29,31^.

In the present study, the formation of highly oxidized products from the reaction of OH radicals with toluene was experimentally investigated for close to atmospheric reaction conditions. This reinvestigation became necessary because of existing discrepancies in the literature regarding the mechanistic understanding of the autoxidation processes as well as in the assessment of the importance of HOM formation from toluene under atmospheric conditions. In addition to our earlier experiments^27,31^, (i) different ionization schemes were applied allowing altogether more reliable HOM measurements, (ii) usage of partly deuterated toluene gave an insight in the internal H-abstraction processes, and (iii) investigations in the presence of NO revealed the HOM formation relevant for atmospheric conditions. Results of further experiments on OH + benzene supported the discussion on the findings in the toluene system.

## Results

The investigations were carried out in a laminar flow tube (LFT)^33^ at 295 ± 0.5 K and a pressure of 1 bar of purified air with a reaction time of 32 s. The relative humidity (r.h.) of the reaction gas was < 0.1% with exception of the H/D exchange experiments applying r.h. of ∼1.5%. OH radicals were produced either via ozonolysis of tetramethylethylene (TME) or by photolysis of isopropyl nitrite (IPN). The NO concentrations ranged from < 2 × 10^8^ molecules cm^-3^, which stand for the background NO level^31^ allowing undisturbed unimolecular RO_2_ reactions or RO_2_ + R´O_2_ steps, up to 5 × 10^11^ molecules cm^-3^ representing conditions in polluted areas characterized by the dominant RO_2_ + NO reaction. Detection of the reaction products was performed using a CI-APi-TOF (chemical ionisation - atmospheric pressure interface - time-of-flight) mass spectrometer with a detection limit down to ∼ 10^4^ molecules cm^-3^ applying a suite of reagent ions, i.e., acetate (CH_3_COO^-^), iodide (I^-^), ethylaminium (C_2_H_5_NH_3_^+^), n-propylaminium (C_3_H_7_NH_3_^+^) and nitrate (NO_3_^-^)^34,35^.

### HOM-RO_2_ radical formation for low-NO conditions

**Fig. 1:**
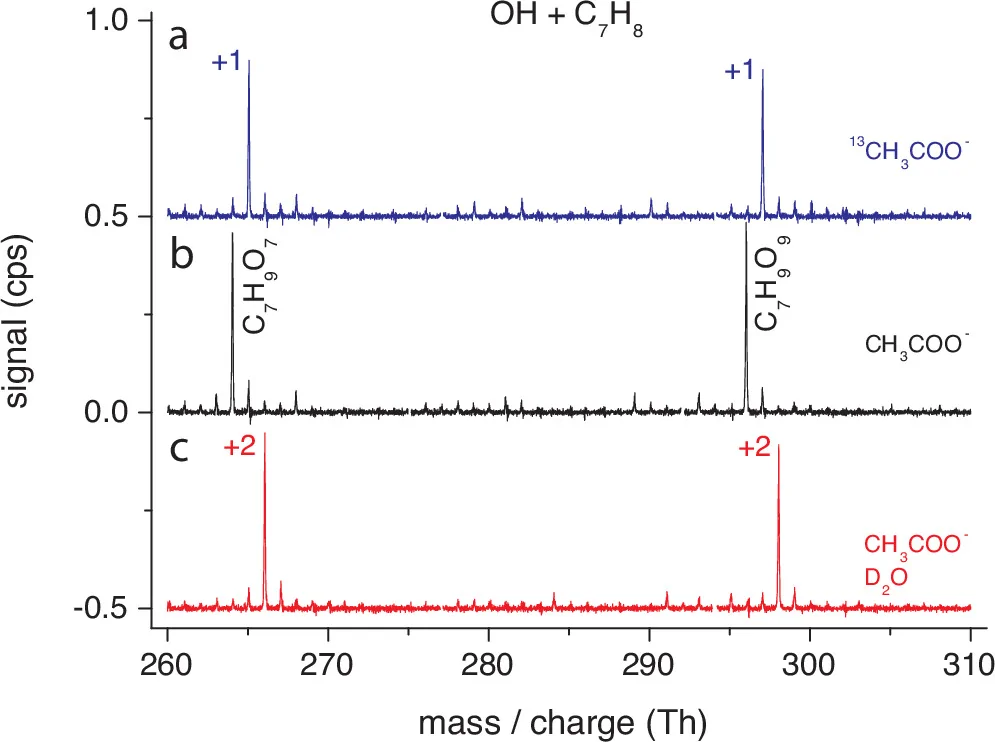
Mass spectra from OH + C_7_H_8_ using CH_3_COO^-^ or ^13^CH_3_COO^-^ for product ionization. Selected range of 260–310 Th shows the signals of formed HOM-RO_2_ radicals which appear as an adduct with the reagent ion. Background signals were removed from the spectra. **a** Measured signals obtained by means of ^13^CH_3_COO^-^ ionization, **b** by CH_3_COO^-^ ionization and **c** by CH_3_COO^-^ ionization in the presence of heavy water for H/D exchange. OH radicals were produced from TME ozonolysis and converted C_7_H_8_ was 7.8 × 10^8^ molecules cm^-3^. The relative humidity in the D_2_O experiment was ∼1.5% not noticeably changing the detection sensitivity compared to dry conditions.

First, the formation of highly oxidized RO_2_ radicals in the OH + toluene reaction was investigated for background NO level in the LFT, i.e., [NO] < 2 × 10^8^ molecules cm^-3^, using OH radical production via TME ozonolysis. Figure 1 shows measured spectra applying CH_3_COO^-^ or ^13^CH_3_COO^-^ ionization. The signal shift by one mass unit in the case of ^13^CH_3_COO^-^ (Fig. 1a) relative to CH_3_COO^-^ (Fig. 1b) revealed that the measured signals were adducts with the reagent ions. Any interference by deprotonation signals of other products or impurities can be neglected^36,37^. In H/D exchange experiments with heavy water (D_2_O), the number of exchangeable H atoms in both RO_2_ radicals was obtained being two each (Fig. 1c). That means for C_7_H_9_O_7_ that, in addition to the -OH group from initial OH attack, most likely an -OOH group exists, which was formed in an autoxidation step after internal H-abstraction of a ring- or side chain-H atom followed by O_2_ addition to the newly formed alkyl position. In the case of the formation of C_7_H_9_O_9_, however, it can be ruled out a further H-abstraction step from the former C_7_H_8_ skeleton, which would result in a next -OOH group with a next exchangeable H atom. Here, the autoxidation could proceed via an endo-cyclization step^38^ or it could be started by internal H-abstraction from the -OH group as proposed by Iyer et al.^32^.

The HOM-RO_2_ data obtained by means of CH_3_COO^-^ ionization were in good agreement with those measured earlier^27^. Again, there was a small signal of a C_7_H_8_O_6_ product detectable. This product was not further considered in the analysis because of its low signal intensity close to the background level.

Results from experiments using NO_3_^-^ ionization qualitatively confirmed the findings using CH_3_COO^-^ ionization, i.e., two exchangeable H atoms in C_7_H_9_O_7_ and C_7_H_9_O_9_ (Supplementary Fig. 1). The ratio of the signal intensity of both HOM-RO_2_ radicals, however, was clearly different, cf. Fig. 1 with Supplementary Fig. 1, indicating different detection sensitivities applying either CH_3_COO^-^ or NO_3_^-^ ionization. In addition, C_7_H_9_O_11_ radicals from a next autoxidation step became visible using NO_3_^-^ ionization, but with low signal intensity. H/D exchange experiments showed two or three exchangeable H atoms in C_7_H_9_O_11_ suggesting the occurrence of different isomeric structures (Supplementary Fig. 1).

For comparison, in the spectra of the OH + C_6_H_6_ reaction measured by means of NO_3_^-^ ionization only the formation of C_6_H_7_O_9_ and C_6_H_7_O_11_ radicals was clearly visible, here again with two and two or three exchangeable H atoms, respectively (Supplementary Fig. 2). The similarity points to comparable processes in the autoxidation of both aromatic compounds.

**Fig. 2:**
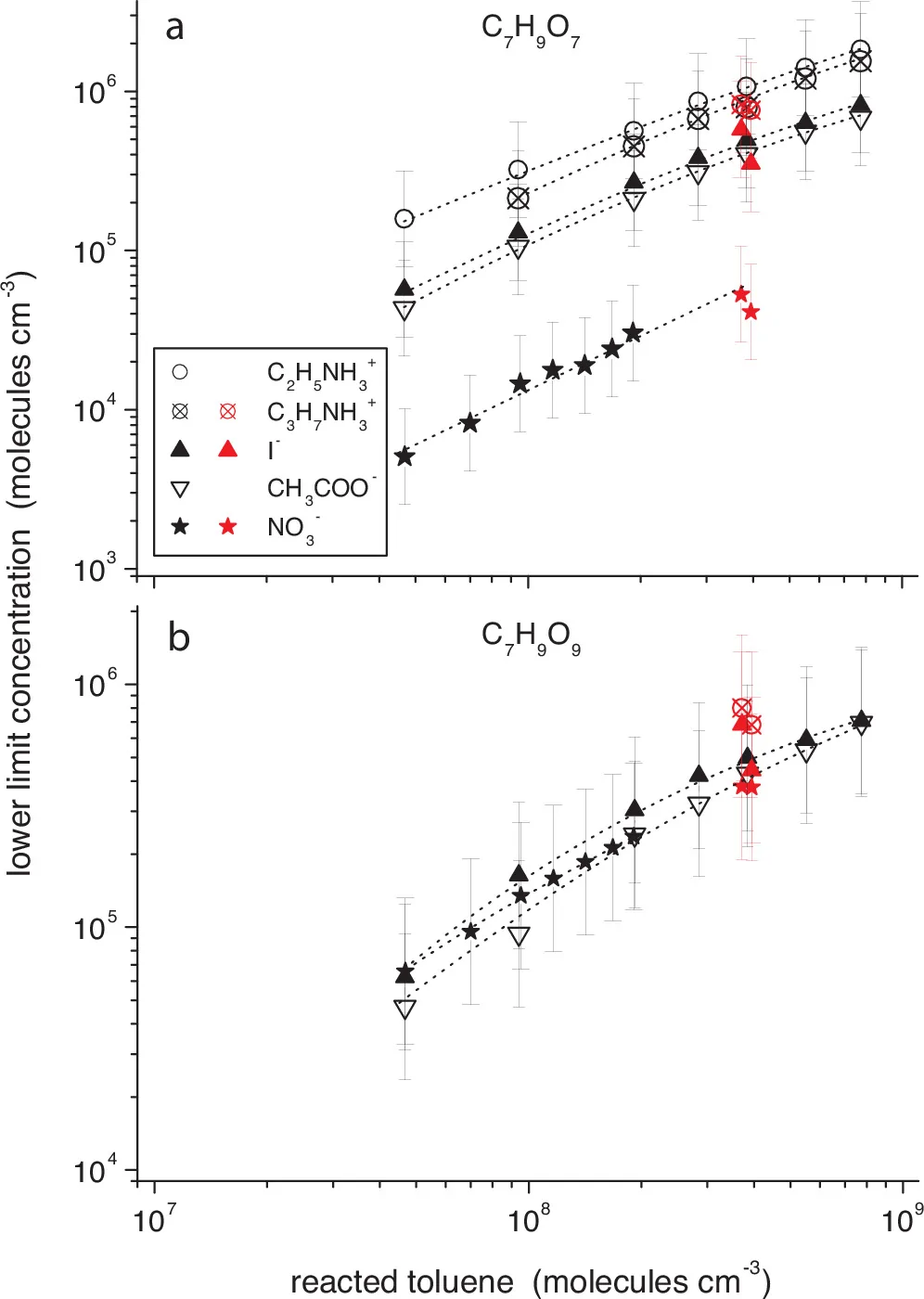
HOM-RO_2_ radical generation from OH + C_7_H_8_ for low-NO conditions. **a** Formation of C_7_H_9_O_7_ and **b** of C_7_H_9_O_9_ applying different ionization schemes. Black symbols represent the results from TME ozonolysis for OH radical generation and the red ones from IPN photolysis for [NO] < 2 × 10^9^ molecules cm^-3^. The error bars represent an uncertainty of a factor of two in the lower limit concentrations based on the uncertainty of the calculated calibration factor. The amount of reacted toluene was calculated by a model in the case of TME ozonolysis (Supplementary Note 1) or otherwise measured by means of the SO_3_ method (Methods). Reactant concentrations were (unit: molecules cm^-3^): [TME] = 2.6 × 10^10^, [O_3_] = (6.2–104) × 10^10^ and [C_7_H_8_] = 4.4 × 10^12^ or for IPN photolysis: [IPN] = 7.7 × 10^10^ and [C_7_H_8_] = 4.4 × 10^12^.

In Fig. 2, experimental results of C_7_H_9_O_7_ and C_7_H_9_O_9_ radical measurements by five ionization schemes are compiled. All these schemes are applicable for detection of products with several functional groups, such as -OH or -OOH^25,34^. Stated concentrations are given as lower limit values (Methods). The lower limit concentrations approach a threshold value with increasing detection sensitivity of the ionization scheme, which should be close to the “real” concentration^34,39^. For C_7_H_9_O_7_, ionization by the aminium ions C_2_H_5_NH_3_^+^ and C_3_H_7_NH_3_^+^ showed the best sensitivity being slightly higher than that of I^-^ and CH_3_COO^-^, but still within the range of uncertainty of a factor of two. On the other hand, NO_3_^-^ ionization was discovered as less sensitive for C_7_H_9_O_7_ detection (Fig. 2a). For C_7_H_9_O_9_, all ionization schemes in the negative mode, i.e., I^-^, CH_3_COO^-^ and NO_3_^-^, yielded almost identical results within the inaccuracy of measurement (Fig. 2b). Here, analysis by the aminium ions was disturbed by signals of an impurity reaction with ozone making reliable C_7_H_9_O_9_ detection impossible. The results from experiments using IPN photolysis for OH radical generation with [NO] < 2 × 10^9^ molecules cm^-3^ confirmed adequately the findings obtained by means of TME ozonolysis, see the red and black symbols in Fig. 2. Hence, the C_7_H_9_O_7_ concentrations measured by aminium ionization and the C_7_H_9_O_9_ data from all negative ionization schemes can be considered as approximate values of the “real” concentrations. Resulting molar formation yields are ${0.25}_{\,-0.13}^{\,+0.25}\, \%$ (C_7_H_9_O_7_) and ${0.12}_{\,-0.06}^{\,+0.12}\, \%$ (C_7_H_9_O_9_) leading to a total HOM-RO_2_ radical yield from OH + C_7_H_8_ of ${0.37}_{\,-0.19}^{\,+0.37}\, \%$, see Supplementary Figs. 3, 4.

It is to be noted at this point, that the less sensitive detection of C_7_H_9_O_7_ radicals by means of NO_3_^-^ ionization (Fig. 2a) is a next example for a critical approach to this ionization scheme for universal, high-sensitivity HOM measurements, see also the discussion on HOM detection in OH + α/β-pinene^40^. The consideration of the results obtained by other ionization schemes, such as acetate, aminium or ammonium ionization, fundamentally helps to prevent possible misinterpretations.

For comparison, the formation of the HOM-RO_2_ radicals C_6_H_7_O_9_ and C_6_H_7_O_11_ from the OH + C_6_H_6_ reaction was measured by means of CH_3_COO^-^ and NO_3_^-^ ionization (Supplementary Fig. 5). The signal of C_6_H_7_O_7_ radicals was very small and scattered making correct analysis difficult. It is to be noted, that the seeming main product C_6_H_7_O_11_ was only detectable applying NO_3_^-^ as the reagent ion, molar C_6_H_7_O_11_ formation yield: ${0.15}_{\,-0.08}^{\,+0.15}\, \%$ (Supplementary Fig. 5). The C_6_H_7_O_9_ signal intensity measured by means of NO_3_^-^ ionization was about a factor of 2.5 higher than observed in the case of CH_3_COO^-^ ionization. The molar formation yield of C_6_H_7_O_9_ radicals is ${0.06}_{\,-0.03}^{\,+0.06}\, \%$ applying the NO_3_^-^ results (Supplementary Fig. 5). Product ionization by the aminium ions did not show significant HOM-RO_2_ signals above background level. Thus, reliable molar yields of HOM-RO_2_ radicals from OH + C_6_H_6_ cannot be stated based on agreeing results of different ionization schemes. From NO_3_^-^ ionization, a total HOM-RO_2_ yield of ${0.21}_{\,-0.11}^{\,+0.21}\, \%$ for OH + C_6_H_6_ follows, while results of the other ionization schemes point to a value of < 0.1% as earlier stated^31^.

### Labeling experiments and mechanistic implication

**Fig. 3:**
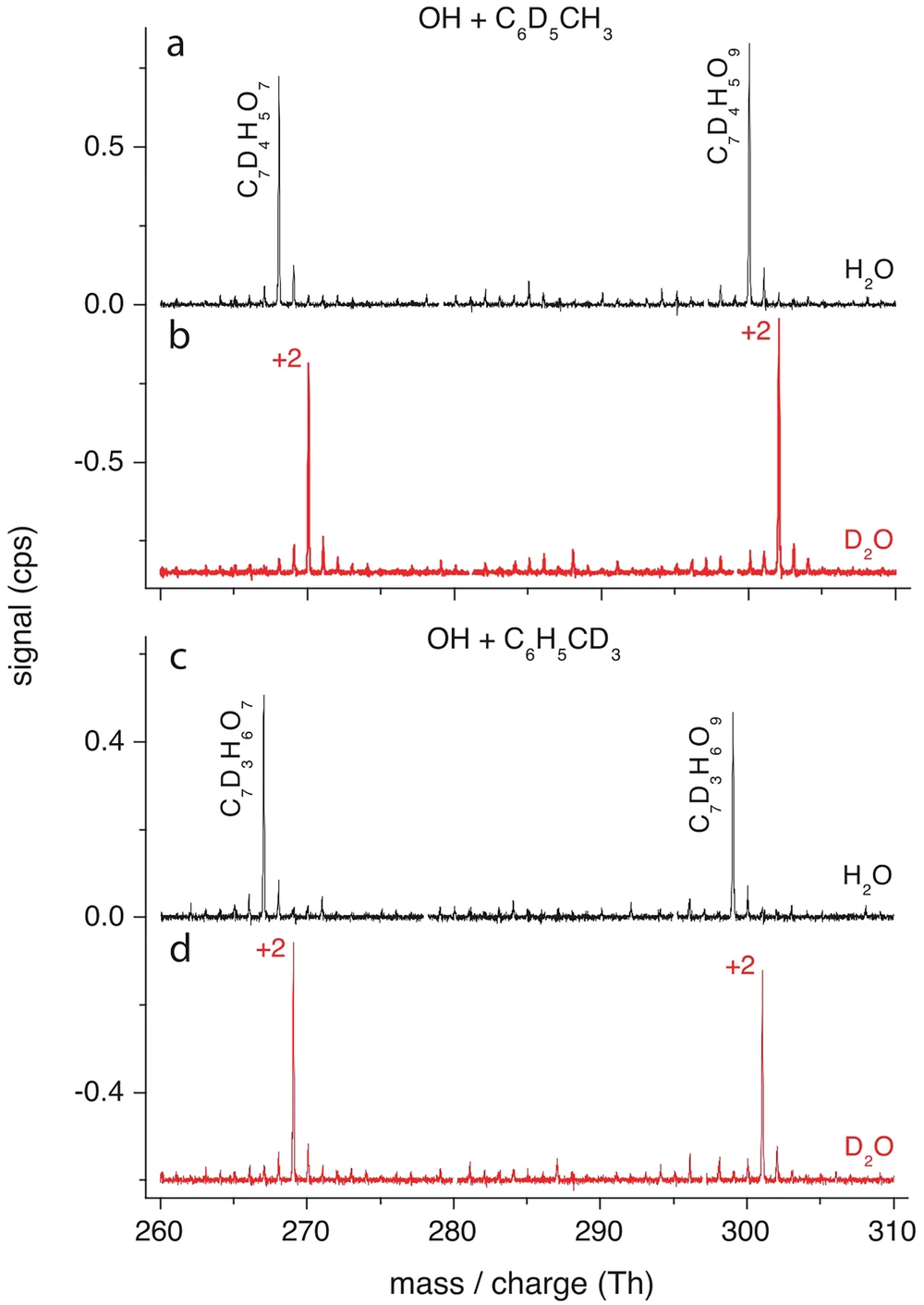
Mass spectra from the OH radical reaction with deuterated toluene using CH_3_COO^-^ for product ionization, low-NO conditions. HOM-RO_2_ radicals appear as an adduct with the reagent ion. Background signals were removed from the spectra. **a** Spectrum from OH + C_6_D_5_CH_3_ (ring deuterated) in the presence of H_2_O and **b** in the presence of D_2_O. **c** Spectrum from OH + C_6_H_5_CD_3_ (side chain deuterated) in the presence of H_2_O and **d** in the presence of D_2_O. OH radicals were produced from the ozonolysis of TME and the amount of reacted toluene was ∼8 × 10^8^ molecules cm^-3^ each. H_2_O or D_2_O addition with a resulting relative humidity of ∼1.5% did not clearly change the sensitivity of the ionization scheme.

Product spectra by means of CH_3_COO^-^ ionization were recorded using ring-deuterated and side chain-deuterated toluene, i.e., C_6_D_5_CH_3_ and C_6_H_5_CD_3_ (Fig. 3). The reaction gas contained either water (H_2_O) or heavy water (D_2_O) with a relative humidity of ∼1.5% allowing to switch between D/H and H/D exchange of exchangeable D or H atoms in the HOM-RO_2_ radicals.

In the presence of H_2_O, in the O_7_ RO_2_ radical from OH + C_6_D_5_CH_3_, C_7_D_4_H_5_O_7_, one D atom less was present than in the reacted toluene, which indicated an autoxidation step by abstraction of a ring-D atom forming most likely a -OOD group (Fig. 3a). Subsequent D/H exchange converted this into a -OOH group. No loss of a D atom in the corresponding O_7_ RO_2_ radical from OH + C_6_H_5_CD_3_, C_7_D_3_H_6_O_7_, confirmed the dominant H(D)-abstraction from the ring and not from the side chain (Fig. 3c). In accordance with the findings from OH + C_7_H_8_ (Fig. 1), all RO_2_ signals were shifted by two mass units when switching from H_2_O to D_2_O due to the presence of two functional groups with exchangeable H atoms each (Fig. 3b, d). This fact was further supported by the same D atom content in the respective O_7_ and O_9_ RO_2_ radicals starting either from C_6_D_5_CH_3_ or C_6_H_5_CD_3_ (Fig. 3a, c).

Thus, the proposed mechanism by Wang et al.^27^, suggesting H-abstraction from the side chain of BCP, cannot be a main route of HOM-RO_2_ radical formation. The preferred H-abstraction from the ring is in line with the mechanistic suggestion by Iyer et al.^32^ although the necessity of an *ipso* OH attack with subsequent ring opening of the BCP cannot be deduced from the present measurements. Moreover, the favored mechanism of C_7_H_9_O_7_ and C_7_H_9_O_9_ generation by Iyer et al.^32^ is in accordance with the occurrence of two functional groups bearing an exchangeable H atom each, i.e., -OH or -OOH, in both HOM-RO_2_ radicals, as observed in the H/D exchange experiments.

In the case of the ring-deuterated toluene, the expected kinetic isotope effect should slow down the formation of the O_7_ RO_2_ radical, which is not visible in the signal intensity of this radical comparing the results from the reaction with C_7_H_8_, C_6_D_5_CH_3_ and C_6_H_5_CD_3_ for similar reactant conversion (Fig. 1b, 3a, c). Any reasoned explanation cannot be given.

### HOM-RO_2_ radical formation proposed by Iyer et al.^32^ and quantitative assessment

**Scheme 2:**
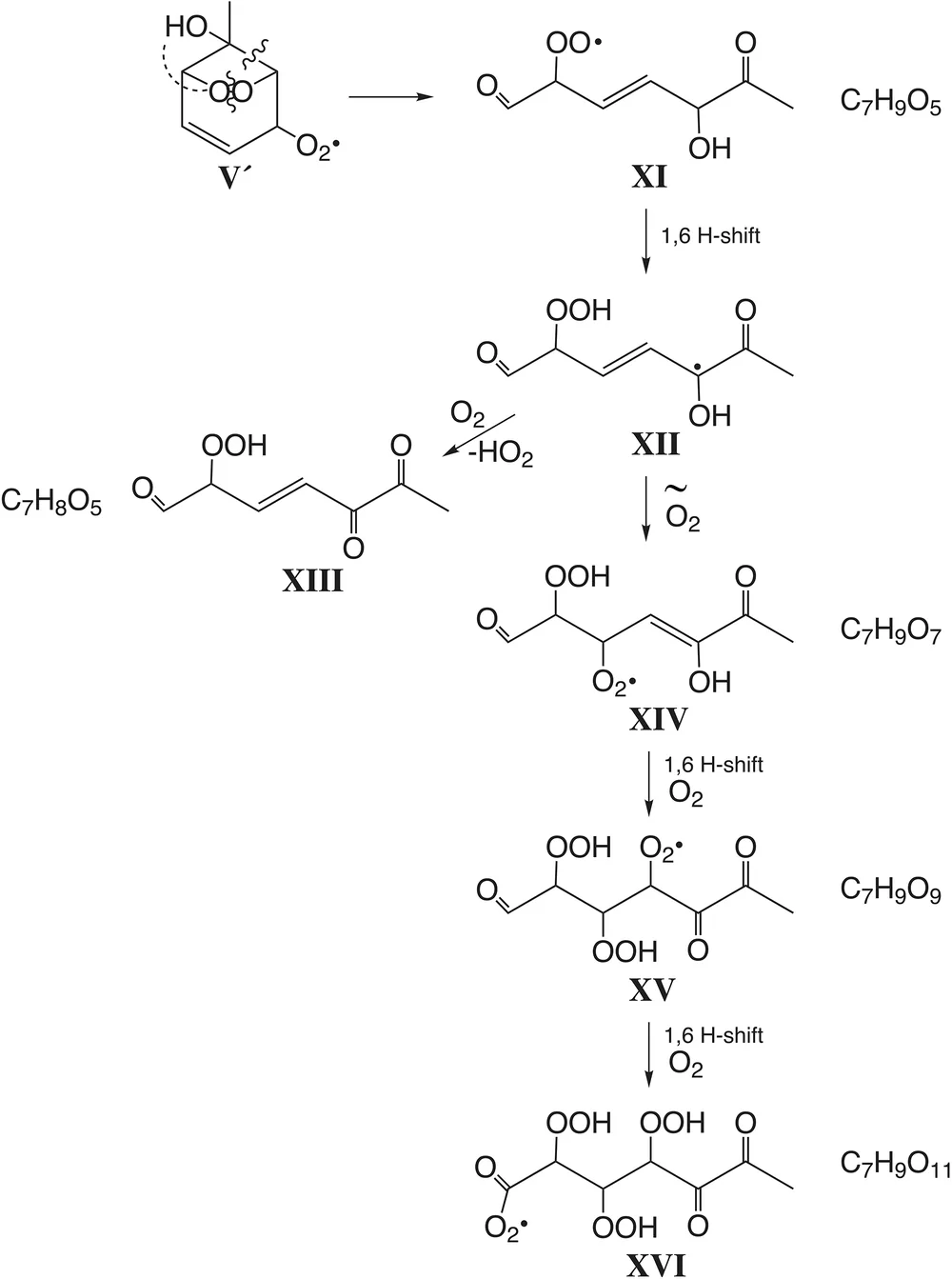
Ring opening of *ipso* BCP from OH + toluene and subsequent reaction steps forming HOM-RO_2_ radicals. The scheme represents the “green route” in a slightly simplified way as given by Iyer et al.^32^. A possible reaction step forming C_7_H_9_O_11_ radicals was added, **XV** → **XVI**.

The qualitative agreement of the experimental findings with the mechanistic suggestion by Iyer et al.^32^ justified to assume a reaction sequence for C_7_H_9_O_7_ and C_7_H_9_O_9_ generation as proposed by Iyer et al.^32^ (Scheme 2). Here, the most favored “green route” starting from the *ipso* BCP is depicted in a simplified way^32^. An additional step starting from C_7_H_9_O_9_ was added in order to explain tentatively the formation of C_7_H_9_O_11_ radicals bearing three exchangeable H atoms, as observed in small quantities. The three autoxidation steps, i.e., **XI** → **XIV, XIV** → **XV** and **XV** → **XVI** (Scheme 2), represent a 1,6 H-shift with subsequent O_2_ addition each.

The preferred ring opening of the *ipso* BCP was calculated to proceed with k(**V´**→ **XI**) = 0.59 s^-1^ at T = 300 K^32^, meaning this process should be practically complete within the reaction time of the experiment of 32 s. Subsequent processes were expected taking place at sub-second scale while the importance of reaction **XII** → **XIII** forming the non-HOM product C_7_H_8_O_5_ was not determined (Scheme 2)^32^. Analysis of the C_7_H_8_O_5_ signal from the low-NO measurements yielded a molar formation yield of ${0.24}_{\,-0.12}^{\,+0.24}\, \%$ for **XIII** presuming correct product assignment (Supplementary Fig. 6). This finding indicates a similar O_2_ reactivity of both allyl positions in **XII** forming either C_7_H_8_O_5_ (**XIII**) or the HOM-RO_2_ radicals. The total product yield of HOM-RO_2_ radicals and C_7_H_8_O_5_ altogether of ${0.61}_{\,-0.31}^{\,+0.61}\, \%$ is a factor of about 6 - 50 lower than expected considering the *ipso* BCP ring opening and subsequent reaction steps as described by Iyer et al.^32^ along with an *ipso* BCP fraction of 7 - 15% from OH + toluene^3,4^. Thus, also the experimentally observed HOM-RO_2_ formation is clearly smaller than expected from the theoretical work by Iyer et al.^32^. A possible reason could be the disregard of further reaction pathways forming non-HOM products after ring opening of the BCP (Scheme 2).

### Accretion products ROOR´

Formation of RO_2_ radical accretion products^25,37^ was only detectable from the BCP self-reaction, pathway (1).

1 $$2{{\rm{HO}}}-{{{\rm{C}}}}_{7}{{{\rm{H}}}}_{8}({{{\rm{O}}}}_{2}){{{\rm{O}}}}_{2}\to {{{\rm{C}}}}_{14}{{{\rm{H}}}}_{18}{{{\rm{O}}}}_{8}+{{{\rm{O}}}}_{2}$$

The best C_14_H_18_O_8_ detection sensitivity was achieved by means of the aminium ions C_2_H_5_NH_3_^+^ and C_3_H_7_NH_3_^+^, which showed almost identical results (Supplementary Fig. 7). Corresponding C_14_H_18_O_8_ concentrations were assumed to be a good approximation of the “real” concentrations such as in the case of other ROOR´ measured by aminium ionization^34,41^. The kinetic analysis yielded an estimate of the room-temperature rate coefficient k_1_ = 1.4 × 10^-12 ^cm^3^ molecule^-1^ s^-1^ with an uncertainty of a factor of 2–3 (Supplementary Fig. 8). Because of the absence of reliable high-sensitivity BCP measurements based on the ionization schemes used in this work, the needed HO-C_7_H_8_(O_2_)O_2_ concentrations were taken from the calculated toluene conversion considering the experimentally observed molar BCP yield of 52%^14,23^. HO-C_7_H_8_(O_2_)O_2_ consumption by bimolecular reactions can be neglected under the experimental conditions, as shown by the results of kinetic analysis, justifying to calculate its concentration from the toluene conversion (Supplementary Note 1). It is to be noted, that k_1_ is about a factor of 100 smaller than the stated rate coefficient of the corresponding mesitylene-derived accretion reaction^41^ where BCP concentrations (HO-C_9_H_12_(O_2_)O_2_) were taken from NH_4_^+^-CI3-TOF measurements^42^. However, using in the re-analysis of the mesitylene system also the calculated BCP concentrations based on a molar BCP yield of ∼85%^14,23^ from OH + C_9_H_12_, the resulting rate coefficient of accretion product (C_18_H_26_O_8_) formation is very similar to k_1_ from the toluene system. It could be speculated that the NH_4_^+^-CI3-TOF measurements significantly underestimated the BCP concentrations^41,42^. Here, more investigations are needed to clarify this inconsistency.

### Product formation in the presence of NO

**Fig. 4:**
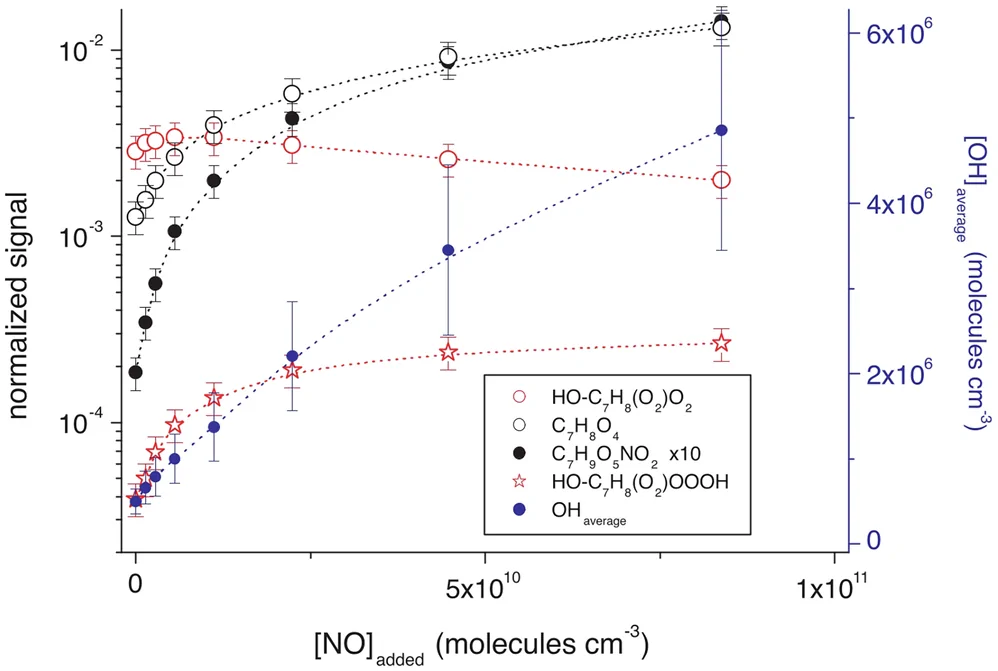
Product formation from OH + C_7_H_8_ and average OH radical concentrations depending on added NO. OH radicals were generated by IPN photolysis. Organic products were measured by I^-^ ionization and OH concentrations by means of the SO_3_ method (Methods). The error bars represent ± 20% uncertainty of the normalized product signals standing for maximum statistical uncertainty of signal intensities for 10 min data accumulation. For OH radical concentrations, the error bars show ± 30% uncertainty of the calibration. Reactant concentrations were (unit: molecules cm^-3^): [IPN] = 7.7 × 10^10^, [C_7_H_8_] = 4.4 × 10^12^ and added [NO] = (1.4–84) × 10^9^.

First, moderate NO additions of (1.4–84) × 10^9^ molecules cm^-3^ were used in the experiments with IPN photolysis for OH radical generation. Rising NO content led to a strong increase of the OH level in the flow tube according to NO + HO_2_ → OH + NO_2_, [OH]_average_ = 5.0 × 10^5^ to 4.9 × 10^6^ molecules cm^-3^, connected with an increase of the toluene conversion from 3.9 × 10^8^ to 3.8 × 10^9^ molecules cm^-3^ (Fig. 4, Supplementary Fig. 9). With rising NO, the BCP signal, HO-C_7_H_8_(O_2_)O_2_, dropped down a bit after a slight initial increase due to overcompensation of its increasing production by the consumption via BCP + NO. The signal of the corresponding hydrotrioxide^43,44^ of the BCP formed via pathway (2)

2 $${{\rm{HO}}}-{{{\rm{C}}}}_{7}{{{\rm{H}}}}_{8}({{{\rm{O}}}}_{2}){{{\rm{O}}}}_{2}+{{\rm{OH}}}\to {{\rm{HO}}}-{{{\rm{C}}}}_{7}{{{\rm{H}}}}_{8}({{{\rm{O}}}}_{2}){{\rm{OOOH}}}$$

became clearly visible for high OH levels. Its signal increased linearly with the product of the BCP signal and the observed OH radical concentration according to its formation kinetics which confirmed the signal assignment. The observed strong C_7_H_8_O_4_ signal followed the product of the BCP signal and the NO concentration indicating that it could be formed from the alkoxy radical **VII** or **VIII** (Scheme 1). Recent studies describe C_7_H_8_O_4_ as the corresponding ketone formed from **VII** supporting this idea^14,45^. However, the C_7_H_8_O_4_ signal was also observed in the low-NO experiments with an apparent constant formation yield. Thus, increase of the C_7_H_8_O_4_ signal with raising NO could be mainly due to rising toluene conversion with raising OH level in the measurements assuming C_7_H_8_O_4_ as a product of OH + toluene without a further RO_2_ + NO reaction. Here, more investigations are needed. Interestingly, the signal of the unexpected organic nitrate C_7_H_9_O_5_NO_2_ appeared which is featured by a very strong NO-dependence (Fig. 4). This behavior points to more than one RO_2_ + NO step needed for its production. The relatively intense signal strength suggests that C_7_H_9_O_5_NO_2_ is mainly formed starting from the BCP **V** and not from C_7_H_9_O_7_. So, C_7_H_9_O_5_NO_2_ formation can be mechanistically explained beginning with **IX**, which is produced from BCP + NO followed by two isomerization steps (Scheme 1). O_2_ addition to the allyl position in **IX** yields the RO_2_ radical C_7_H_9_O_6_ that forms C_7_H_9_O_5_NO_2_ in the reaction with NO:

3 $${{{\rm{C}}}}_{7}{{{\rm{H}}}}_{9}{{{\rm{O}}}}_{4}({{\bf{I}}}{{\bf{X}}})+{{{\rm{O}}}}_{2}\to {{{\rm{C}}}}_{7}{{{\rm{H}}}}_{9}{{{\rm{O}}}}_{6}$$

4 $${{{\rm{C}}}}_{7}{{{\rm{H}}}}_{9}{{{\rm{O}}}}_{6}+{{\rm{NO}}}\to {{{\rm{C}}}}_{7}{{{\rm{H}}}}_{9}{{{\rm{O}}}}_{5}{{{\rm{NO}}}}_{2}$$

**Fig. 5:**
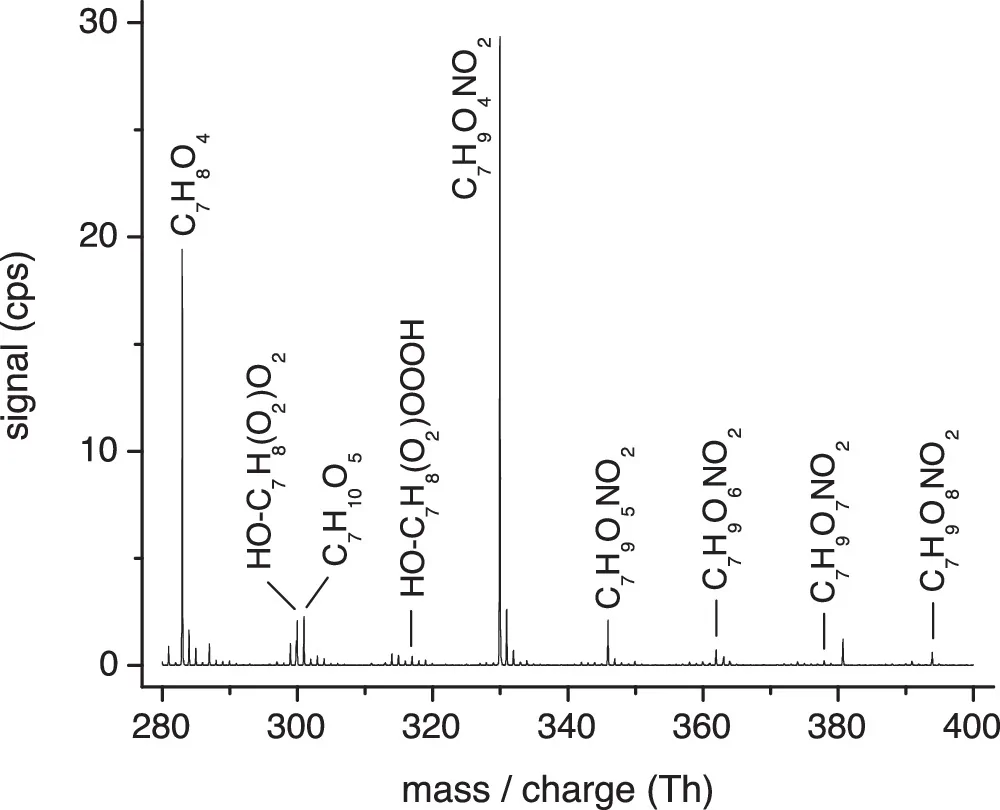
Mass spectrum from OH + C_7_H_8_ in the presence of NO applying I^-^ ionization. Product signals appear as adduct with the reagent ion. The selected range of 280-400 Th depicts detected C_7_ products with at least four O atoms. The signal at 380.71 Th stands for I_3_^-^. Reactant concentrations were (unit: molecules cm^-3^): [IPN] = 7.7 × 10^10^, [C_7_H_8_] = 4.4 × 10^12^ and added [NO] = 8.4 × 10^10^ with a toluene conversion of 3.8 × 10^9^ molecules cm^-3^.

Figure 5 shows the product spectrum in the range of C_7_ products with at least four O atoms recorded for added [NO] = 8.4 × 10^10^ molecules cm^-3^ using I^-^ ionization. Besides the already discussed products and C_7_H_10_O_5_, which is likely the hydroperoxide from the BCP + HO_2_ reaction, only organic nitrates could be clearly identified in this mass range. C_7_H_9_O_4_NO_2_ is attributed to the BCP + NO reaction and C_7_H_9_O_6,8_NO_2_ to the NO reaction of both HOM-RO_2_ radicals. The formation of C_7_H_9_O_7_NO_2_ can be explained starting from both HOM-alkoxy radicals considering a next RO_2_ + NO step after isomerization. The analysis of the organic nitrates based on I^-^ and C_3_H_7_NH_3_^+^ ionization yielded almost identical results. Measured concentrations from these ionization schemes can be assumed to be a good approximation of the “real” concentrations (Supplementary Fig. 10). The absence of other substantial product signals in this mass range indicates preferred decomposition of the HOM-alkoxy radicals. Mass spectra in the lower mass range showed the occurrence of a couple of signals which are at least partly consistent with C_4_- and C_5_-products already described in the literature, e.g., references^14,45^.

**Fig. 6:**
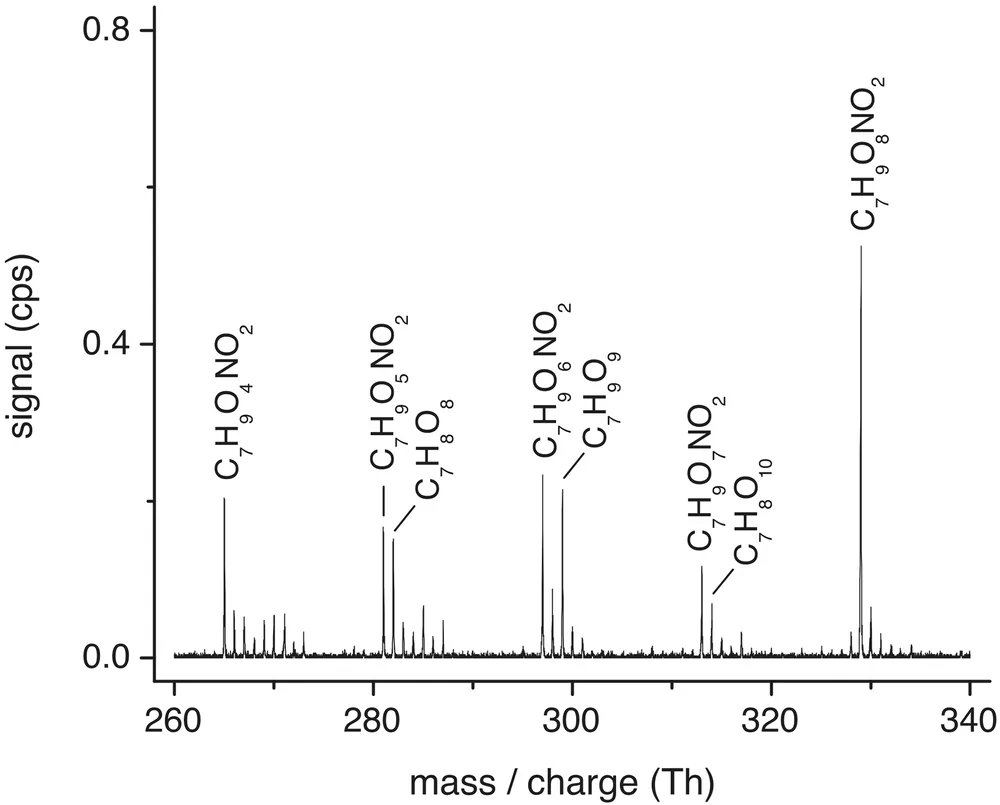
Mass spectrum from OH + C_7_H_8_ in the presence of NO using NO_3_^−^ ionization. Product signals appear as adduct with the reagent ion. The experimental conditions are identical with those used for recording the I^-^ spectrum given in Fig. 5.

The corresponding spectrum measured by means of NO_3_^-^ ionization, which is frequently used for selective HOM detection^25^, shows besides the five organic nitrates also small signals of C_7_H_8_O_8_ and C_7_H_8_O_10_ as well as residual C_7_H_9_O_9_ radicals (Fig. 6). Both non-nitrate closed-shell HOMs were hard to detect accurately using I^-^ and C_3_H_7_NH_3_^+^ ionization due to their small signal intensities and the relative strong isotope signals of the organic nitrates in the immediate vicinity. Any mechanistic explanation of formation routes to C_7_H_8_O_8,10_ is highly speculative based on the current experimental information. With exception of C_7_H_9_O_7,8_NO_2_, the NO_3_^-^ ionization scheme was clearly less sensitive for organic nitrate detection compared to I^-^ and C_3_H_7_NH_3_^+^ ionization.

For comparison, analysis in the OH + C_6_H_6_ system in the presence of NO revealed a similar product pattern as observed in the toluene system. Here, C_6_H_6_O_4_ appeared as the strongest signal in the spectrum recorded by means of I^-^ ionization followed by C_6_H_7_O_4,5_NO_2_ as well as traces of the expected signal of the hydrotrioxide HO-C_6_H_6_(O_2_)OOOH and C_6_H_7_O_6_NO_2_ (Supplementary Fig. 11). Higher oxidized organic nitrates were only measured using NO_3_^-^ ionization (Supplementary Fig. 12). Other closed-shell HOMs were not detectable, not even in traces.

### Molar product yields for extended NO range

**Fig. 7:**
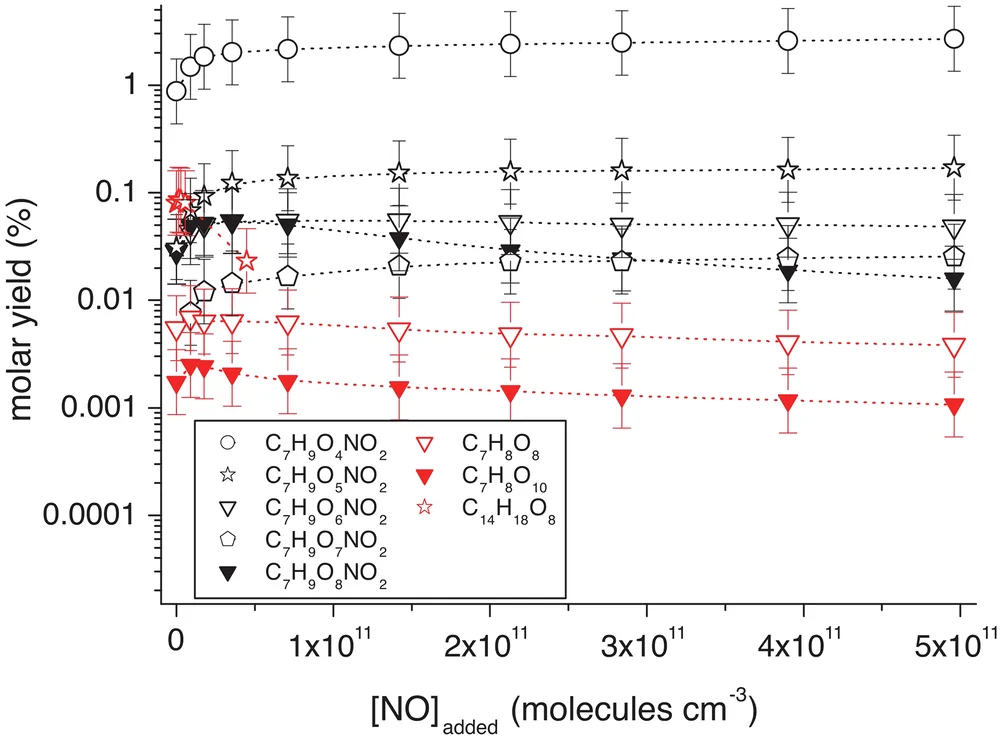
Molar formation yields of closed-shell HOMs and C_7_H_9_O_4_NO_2_ from OH + C_7_H_8_ for an extended range of NO additions. OH radicals were generated by IPN photolysis. Organic nitrates were measured by means of I^-^, C_14_H_18_O_8_ by C_3_H_7_NH_3_^+^ and C_7_H_8_O_8,10_ by NO_3_^-^ ionization. Reacted C_7_H_8_ was determined using the SO_3_ method (Methods). Error bars represent the uncertainty of a factor of two based on the uncertainty of the calculated calibration factor. Reactant concentrations were (unit: molecules cm^-3^): [IPN] = 7.8 × 10^10^, [C_7_H_8_] = 4.4 × 10^12^ and added [NO] = (8.8–495) × 10^9^.

Experiments were repeated for an extended range of NO concentrations of up to 5 × 10^11^ molecules cm^-3^ covering the situation in highly polluted areas such as in megacities. Here, the OH radical concentrations ranged from 4.7 × 10^5^ to 5.7 × 10^6^ molecules cm^-3^ with a toluene conversion of 3.7 × 10^8^ up to 4.5 × 10^9^ molecules cm^-3^. Molar formation yields of organic nitrates, the accretion product C_14_H_18_O_8_ as well as C_7_H_8_O_8_ and C_7_H_8_O_10_ are depicted in Fig. 7. For C_7_H_9_O_4,5_NO_2_, the molar yields became apparently independent of NO for [NO] ≥ 2 × 10^10^ molecules cm^-3^ for the experimental conditions with continuous product generation within the reaction time of 32 s. A molar yield of ${2.5}_{\,-1.3}^{\,+2.5}\, \%$ followed for C_7_H_9_O_4_NO_2_ **X** formed from BCP + NO (Scheme 1). This value is in reasonable agreement with the data given in the literature, 1.2 – 1.6%^14^ and 2.9%^23^. For C_7_H_9_O_5_NO_2_, a molar yield of ${0.16}_{\,-0.08}^{\,+0.16}\, \%$ can be stated. It should be noted that C_7_H_9_O_5_NO_2_, most likely formed from **IX** via reactions (3) and (4), represents the most abundant closed-shell HOM considering its precursor RO_2_ radical C_7_H_9_O_6_ as a next HOM-RO_2_ radical. The molar yield of C_7_H_9_O_6_NO_2_ only slightly declined with rising NO. That implies that the autoxidation step forming C_7_H_9_O_7_ was only slightly repressed even for the highest NO concentration with a bimolecular RO_2_ reactivity of 4.4 s^-1^, k(RO_2_ + NO) = 8.8 × 10^-12 ^cm^3^ molecule^-1^ s^-1^^ 46^. On the other hand, the formation of C_7_H_9_O_9_, and subsequently C_7_H_9_O_8_NO_2_, was strongly pushed back by the RO_2_ + NO reaction indicating a clearly less rapid autoxidation step for C_7_H_9_O_9_ production as proposed for the “green route” by Iyer et al.^32^ (Fig. 7). The total molar yield of C_7_H_9_O_6-8_NO_2_ amounted to ≤ 0.1% in the whole range of NO concentrations. As expected from their production routes, the yields of the accretion product C_14_H_18_O_8_ and the BCP-derived hydrotrioxide HO-C_7_H_8_(O_2_)OOOH distinctly dropped down with rising NO addition making them less important as HOM products. For C_7_H_8_O_8_ and C_7_H_8_O_10_, molar formation yields < 0.01% were obtained assuming maximum detection sensitivity in the NO_3_^-^ ionization. Hence, these both HOMs represent only trace products. For comparison, a molar C_7_H_8_O_8_ yield of about 0.015% can be derived from initial product formation in a chamber study for [NO] = 5 × 10^9^ molecules cm^-3^^ 30^ being in reasonable agreement with the C_7_H_8_O_8_ yield of 0.006 - 0.007% (uncertainty: factor two) for added [NO] < 5 × 10^10^ molecules cm^-3^ from the present study (Fig. 7).

**Fig. 8:**
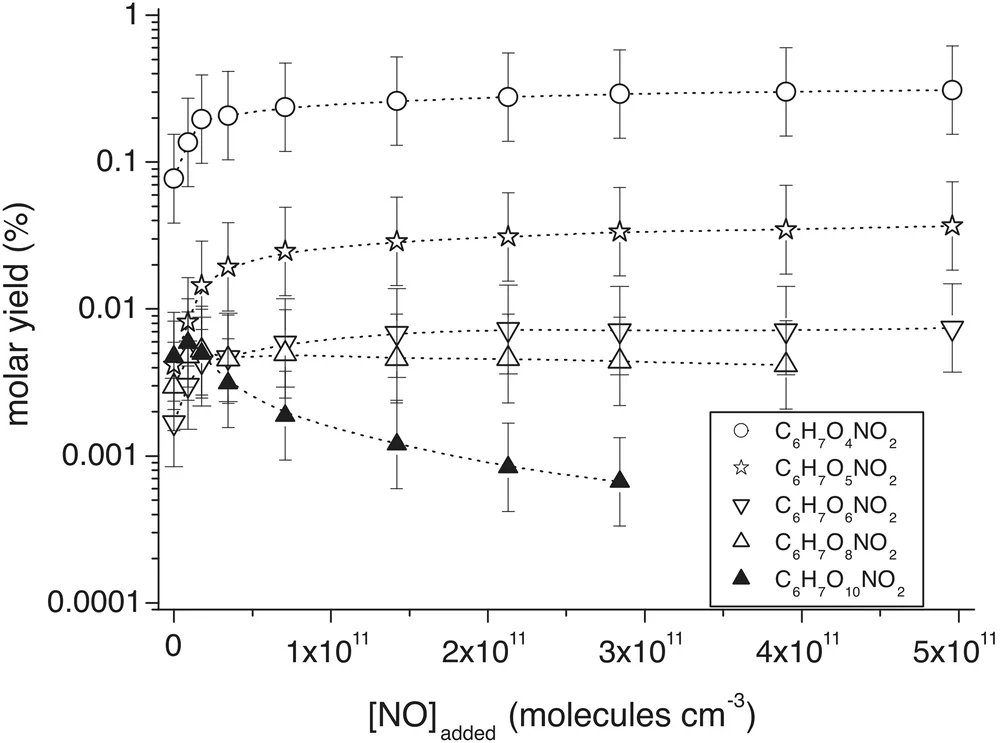
Molar formation yields of organic nitrates from OH + C_6_H_6_ depending on NO additions. OH radicals were generated by IPN photolysis. The C_6_H_7_O_4-6_NO_2_ were measured by means of I^-^ ionization and C_6_H_7_O_8,10_NO_2_ by NO_3_^-^ ionization. Reacted C_6_H_6_ was determined using the SO_3_ method (Methods). The error bars show the uncertainty of a factor of two based on the uncertainty of the calculated calibration factor. Reactant concentrations were (unit: molecules cm^-3^): [IPN] = 7.8 × 10^10^, [C_6_H_6_] = 2.1 × 10^13^ and added [NO] = (8.8–495) × 10^9^.

Figure 8 shows the molar yields of organic nitrates C_6_H_7_O_4-6,8,10_NO_2_ from OH + C_6_H_6_ in the extended range of NO concentrations. With exception of C_6_H_7_O_4_NO_2_, all other organic nitrates can be regarded as HOMs. Other closed-shell HOM products were not detectable. The BCP-derived organic nitrate C_6_H_7_O_4_NO_2_ was measured with a molar formation yield of ${0.28}_{\,-0.14}^{\,+0.28}\, \%$ being higher than the recently reported value of < 0.1%^7^ but clearly smaller than 3.4%^23^ from a former study. For C_6_H_7_O_5_NO_2_, which can be considered as most important HOM in the benzene system, a molar formation yield of ${0.033}_{\,-0.017}^{\,+0.033}\, \%$ follows. Higher oxidized organic nitrates occurred with molar yields of < 0.01%.

## Discussion

Within the present study, the formation of highly oxidized products from the gas-phase reaction of OH radicals with toluene and benzene has been investigated for close to atmospheric reaction conditions focusing on the toluene system.

For low-NO conditions, the HOM-RO_2_ radicals C_7_H_9_O_7_ and C_7_H_9_O_9_ were detected in the OH + toluene reaction with molar yields of ${0.25}_{\,-0.13}^{\,+0.25} \%$ and ${0.12}_{\,-0.06}^{\,+0.12} \%$, respectively. This finding is qualitatively in line with former observations^27,28,31^. Labeling experiments using ring-deuterated and side chain-deuterated toluene revealed the ring H(D) atoms being involved in the autoxidation steps leading to HOM-RO_2_ radicals. Thus, proposed internal H-abstraction from the side chain^27^ can only represent a minor process.

Closed-shell HOM products of the reaction of C_7_H_9_O_7_ and C_7_H_9_O_9_ radicals with NO are almost exclusively the corresponding organic nitrates, which points to the preferred decomposition of the HOM-alkoxy radicals forming non-HOM C_4_- and C_5_-products amongst others as experimentally observed. The total molar formation yield of C_7_H_9_O_6-8_NO_2_ was measured to be ≤ 0.1%. The most important HOM product represents C_7_H_9_O_5_NO_2_ with a molar yield of ${0.16}_{\,-0.08}^{\,+0.16}$ % generated most likely from BCP + NO after C_7_H_9_O_6_ radical formation followed by a next NO reaction. Other closed-shell HOM products, i.e., the hydrotrioxide HO-C_7_H_8_(O_2_)OOOH, the accretion product C_14_H_18_O_8_ from BCP self-reaction and C_7_H_8_O_8,10_, are trace products under dominating RO_2_ + NO conditions.

Results of analog experiments for OH + benzene supported qualitatively the findings from the OH + toluene reaction but showed even smaller formation yields. Here, only organic nitrates were detected as closed-shell HOM products.

All in all, the outcome of this study indicates a minor importance of the OH radical reaction of toluene and benzene for HOM generation under atmospheric conditions. Hence, atmospheric oxidation of toluene does not represent a key route to HOMs and subsequently to HOM-driven aerosol formation as recently proposed^32^.

## Methods

### Experimental setup

The experiments were carried out in a laminar flow tube (LFT)^33^ at 1 bar of air, a temperature of 295 ± 0.5 K and a relative humidity < 0.1% in most cases. In D/H and H/D exchange experiments the reaction gas contained either water (H_2_O) or heavy water (D_2_O) with a relative humidity of ∼1.5% The reaction time in the LFT was 32 s experimentally obtained by a “chemical clock”^47^. Ozonolysis of tetramethylethylene (TME)^48^ or photolysis of isopropyl nitrite (IPN)^49^ served as the OH radical source. IPN photolysis starts with the photolysis step C_3_H_7_ONO → C_3_H_7_O + NO followed by C_3_H_7_O + O_2_ → C_3_H_6_O + HO_2_. OH generation finally proceeds via HO_2_ + NO → OH + NO_2_ accelerated by added NO.

The laminar flow tube (i.d. 8 cm; total length 425 cm) consists of a first section (56 cm) containing the gas inlet system, a second middle section (344 cm) representing the reaction zone surrounded by 24 Philips 36 W Blacklight Blue lamps used for IPN photolysis. An end section (25 cm) incorporates the sampling devices. Applying TME ozonolysis, ozone was injected through the nozzle inlet into the gas mixture, including TME and the aromatics, just before entering the middle section. In the case of photolysis, IPN was added through the nozzle inlet to the main gas stream containing the aromatics and additional NO if needed. The total flow was set at 30 L min^-1^ (STP).

The concentration of the aromatics was monitored by means of a proton transfer reaction mass spectrometer at the outflow of the flow systems (Ionicon, PTR-MS 500)^50^. The relative humidity of the reaction gas was continuously controlled by a humidity sensor (Hygrosense HYTE) and ozone was measured by a gas monitor (Thermo Scientific, iQ 49). All gas flows were set by calibrated gas flow controllers (MKS 1259/1179). Air was taken from a commercial PSA (Pressure Swing Adsorption) unit with further purification by a series of absorber units filled with hopcalite (CuMnOx catalyst), activated charcoal and different 4 Å and 10 Å molecular sieves.

Diffusion-controlled wall loss in the LFT was considered by ${{{\rm{k}}}}_{{{\rm{loss}}}}$ = $\frac{3.65{{\rm{x\; D}}}}{\,{{{\rm{r}}}}^{2}}$ using an average diffusion coefficient for all oxidation products D = 0.08 cm^2 ^s^-1^^ 51^ leading to ${{{\rm{k}}}}_{{{\rm{loss}}}}$ = 0.018 s^-1^^ 39^. Based on that and for the reaction time of 32 s, product loss to the walls of 24% was obtained. The measured product concentrations were corrected accordingly.

### Mass spectrometric analysis

Detection of the oxidation products was carried out using a CI-APi-TOF (chemical ionisation - atmospheric pressure interface - time-of-flight) mass spectrometer with a resolving power > 3000 Th/Th (Tofwerk) connected to a Boulder-type inlet system (Airmodus) operating at atmospheric pressure. Ethylaminium (C_2_H_5_NH_3_^+^), propylaminium (C_3_H_7_NH_3_^+^), acetate (CH_3_COO^-^), iodide (I^-^) and nitrate (NO_3_^-^) served as reagent ions in the product measurements.

In the case of ionization by the aminium ions, the respective amine premixed in a flask was added to a 35 L min^-1^ (STP) sheath flow of high-purity nitrogen resulting in an amine concentration of (2–3) × 10^11^ molecules cm^-3^. Formed reagent ions after ionization by a ^241^Am source were amine-H^+^ and to a lesser amount (amine)amine-H^+^ clusters. The ions from the sheath flow were guided into the sample flow by an electric field without mixing of both gas streams. For ionization by acetate, a flow of 1 ml min^-1^ (STP) nitrogen over high purity acetic acid was added to the sheath flow resulting in CH_3_COO^-^ and (CH_3_COOH)CH_3_COO^-^ formation after ionization. In the case of iodide, tert-butyl iodide premixed in a flask was added to the 35 L min^-1^ (STP) sheath flow leading to a tert-C_4_H_9_I concentration of 5 × 10^11^ molecules cm^-3^. Resulting reagent ions after ionization were I^-^ and small traces of I(H_2_O)^-^. In the case of ionization by nitrate, an HNO_3_ containing vial was connected to the sheath flow without overflowing the HNO_3_ sample. HNO_3_ diffusion from the vial was found to be sufficient to form the reagent ions (HNO_3_)_x_NO_3_^-^, x = 0, 1, 2, after ionization.

All products were detected as adducts with the respective reagent ion, (product)reagent ion. The measured signal intensity of these adducts, [(product)reagent ion], was normalized by dividing by the sum of the signal intensities of all reagent ions of the ionization scheme, Σ[reagent ion], yielding the stated normalized product signals, see Eq. (5).

5 $${{\rm{Normalized}}}\; {{\rm{product}}}\; {{\rm{signal}}}=\frac{\left[\left({{\rm{product}}}\right){{\rm{reagent}}}\; {{\rm{ion}}}\right]}{\sum \left[{{\rm{reagent}}}\; {{\rm{ion}}}\right]}$$

Duty cycle correction is applied in order to compensate for the mass-dependent transmission of the mass spectrometer^34^. Stated results were obtained from 10 min data accumulation. Statistical uncertainty of signal intensities was less than 20%, in most cases less than 10%.

Product concentrations can be obtained from the normalized product signals multiplied by the calibration factor. The calculated calibration factors for lower limit concentrations are in the range f_calc_ = (1.3-2.8) × 10^9^ molecules cm^-3^ assuming i) collision limit in the ion-molecule reaction and ii) no ion loss in the instrument^34^. Here, f_calc_ = 2.3 × 10^9^ molecules cm^-3^ with an uncertainty of a factor of two was used in accordance with the current absolute H_2_SO_4_ calibration using nitrate ionization where requirements i) and ii) are most likely fulfilled.

### Determination of reacted toluene or benzene and the average OH concentration

The conversion of the aromatics in the experiments was smaller than ∼ 0.1% making reliable measurements of reacted aromatics impossible. Hence, the amount of reacted toluene and benzene in the case of TME ozonolysis for OH radical generation was calculated based on a reaction mechanism (Supplementary Note 1). In the case of OH radical production from IPN photolysis, in additional experiments, SO_2_ was added in small quantities not disturbing the product formation from OH + toluene or benzene. From the measured SO_3_ formation measured by means of NO_3_^-^ ionization, OH + SO_2_ ( + O_2_) → SO_3_ + HO_2_, k(295 K) = 8.9 × 10^-13 ^cm^3^ molecules^-1^ s^-1^^52^, the amount of converted aromatic compounds in the LFT was determined, which allowed to calculate the average OH radical concentration, called SO_3_ method^44^. See Supplementary Fig. 9 as an example.

### Kinetic analysis

*ROOR formation from BCP self-reaction:* BCP concentrations in the experiment for low-NO conditions are controlled by their formation step and BCP consuming pathways are negligible. Moreover, the BCP formation rates are practically constant in the course of the reaction because of practically constant reactant concentrations including the OH level.

Thus, the BCP concentrations increase linearly with time and can be written as follows:

6 $$\left[{{\rm{BCP}}}\right]={{\rm{f}}}\left({{\rm{t}}}\right)={{\rm{a}}}\times {{\rm{t}}},{{\rm{a}}}={{\rm{constant}}}$$

The resulting differential equation describing the time dependence of ROOR formation according to Eq. (1) is:

7 $${{\rm{d}}}\left[{{\rm{ROOR}}}\right]/{{\rm{dt}}}={{{\rm{k}}}}_{1}\times \left[{{\rm{BCP}}}\right]\times \left[{{\rm{BCP}}}\right]={{{\rm{k}}}}_{1}\times {{{\rm{a}}}}^{2}\times {{{\rm{t}}}}^{2}$$

Integration of Eq. (7) for [ROOR]_t=0_ = 0 leads to:

8 $${\left[{{\rm{ROOR}}}\right]}_{{{\rm{t}}}}={{{\rm{k}}}}_{1}\times {{{\rm{a}}}}^{2}\times {{{\rm{t}}}}^{3}/3={{{\rm{k}}}}_{1}\times {\left[{{\rm{BCP}}}\right]}_{{{\rm{t}}}}^{2}\times {{\rm{t}}}/3$$

The slope of [ROOR]_t_ vs. [BCP]_t_^2^ yields the rate coefficient k_1_ multiplied by one third of the reaction time, t = 32 s.

## Supplementary information

Supplementary Information

## Data Availability

The experimental data generated in this study are provided in the figures as shown in the paper and in the Supplementary Information. Data are available from the author upon reasoned request.
