# Supplementary material for: Highly oxidized products from the atmospheric reaction of hydroxyl radicals with toluene
Source: Commun Chem. 2026 Oct 8;9:318. doi: 10.1038/s42004-026-02230-0 (PMC13649766; doi:10.1038/s42004-026-02230-0)
Supplement: Supplementary file 1 [file 42004_2026_2230_MOESM1_ESM.pdf]

Supplementary Information for

Highly oxidized products from the atmospheric reaction of hydroxyl radicals with toluene

Torsten Berndt

Atmospheric Chemistry Department (ACD), Leibniz Institute for Tropospheric Research (TROPOS), 04318 Leipzig, Germany.

Corresponding author: Torsten Berndt, e-mail: berndt@tropos.de

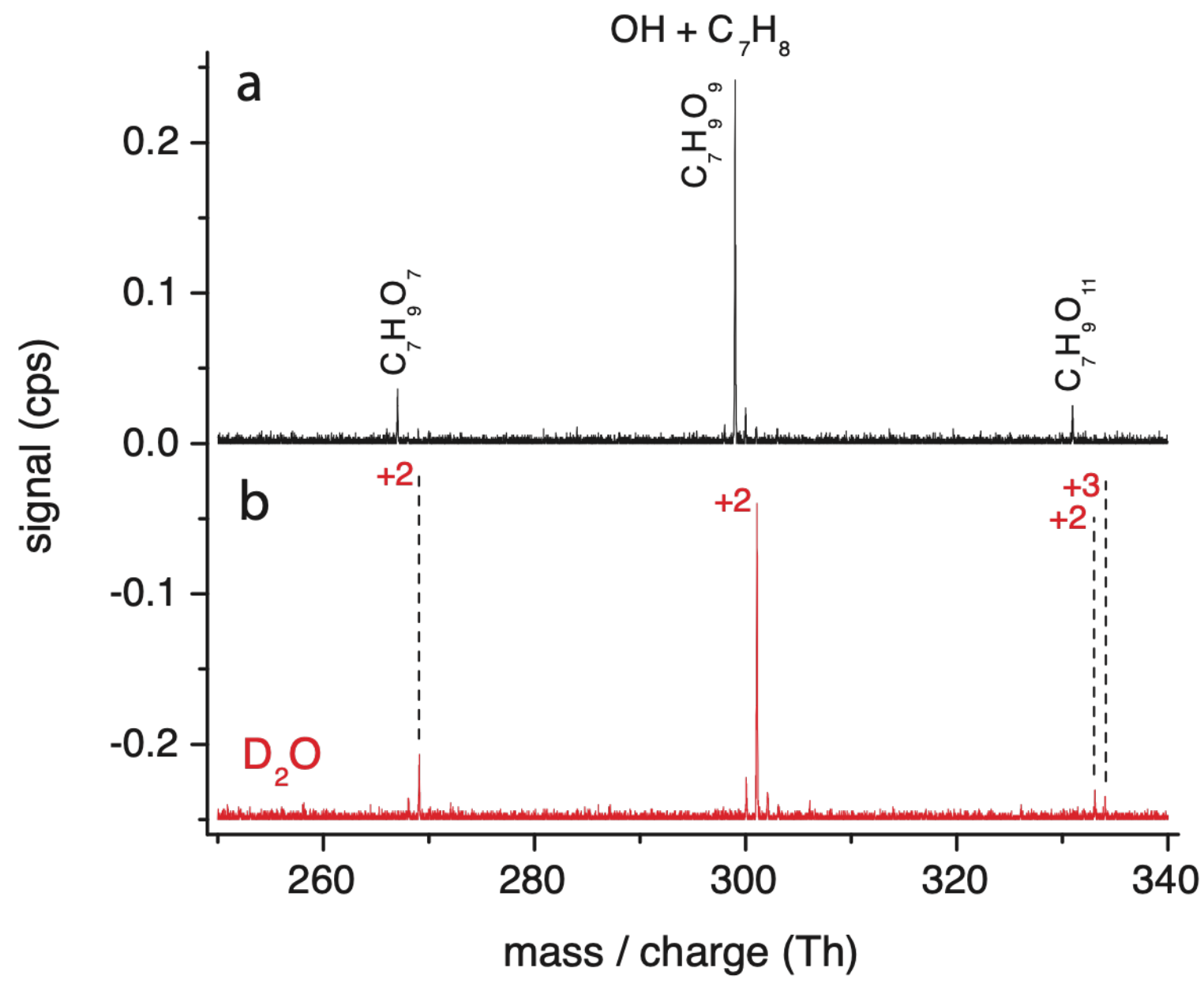

Supplementary Fig. 1 Mass spectra from OH + C_7H_8 using NO_3^- for product ionization. Selected range of 250 - 340 Th shows the signals of formed HOM-RO_2 radicals which appear as an adduct with the reagent ion. **a)** Measured signals obtained in the absence of heavy water and **b)** in the presence of heavy water for H/D exchange. Reacted C_7H_8 was 1.9×10^8 molecules cm^{-3} and OH radicals were produced via TME ozonolysis.

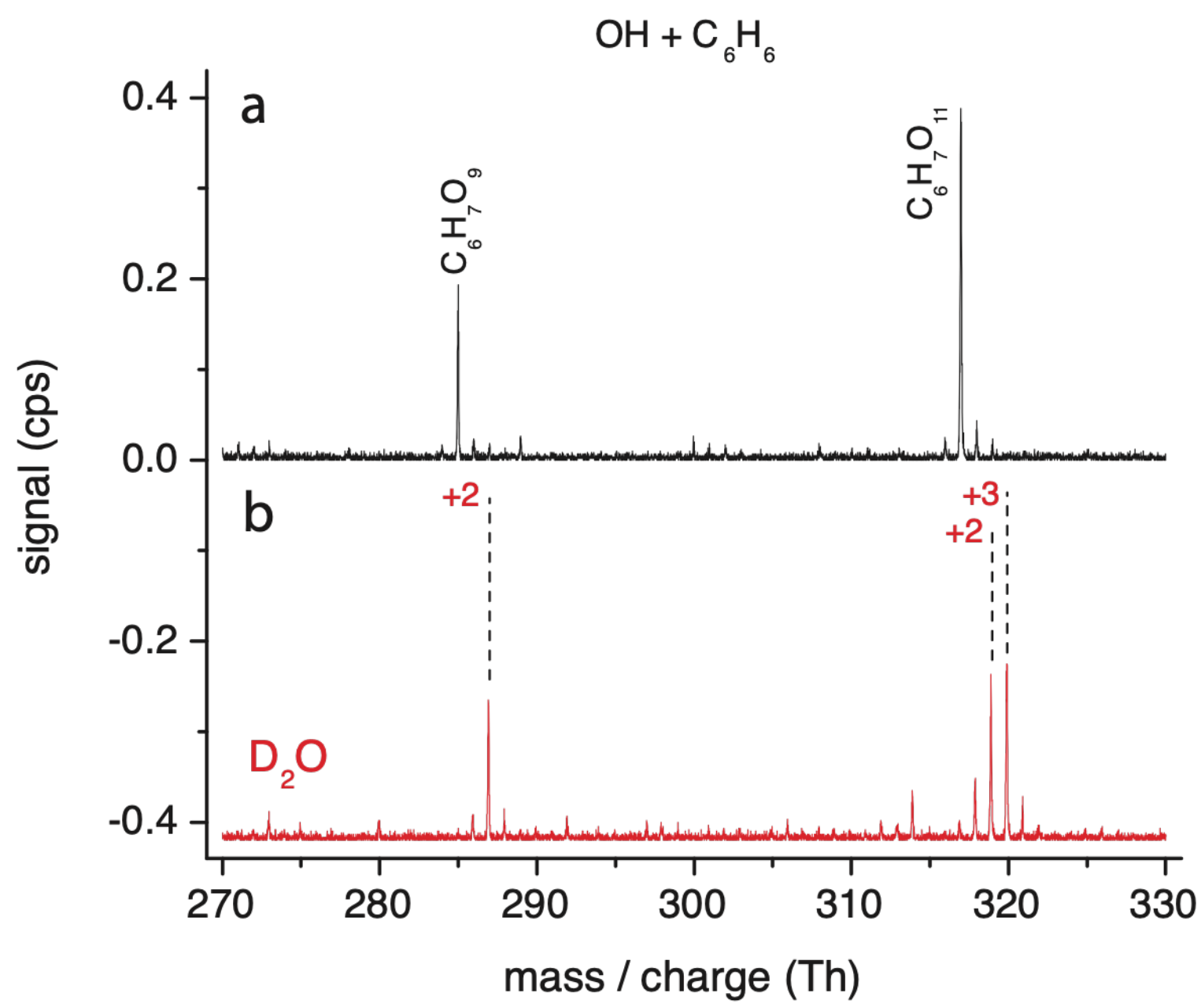

Supplementary Fig. 2 Mass spectra from OH + C_6H_6 using NO_3^- for product ionization. Selected range of 270 - 330 Th shows the signals of formed HOM-RO_2 radicals which appear as an adduct with the reagent ion. **a)** Measured signals obtained in the absence of heavy water and **b)** in the presence of heavy water for H/D exchange. Signals at nominal 314 Th and 318 Th are most likely due to background impurities. Reacted C_6H_6 was 3.4×10^8 molecules cm^{-3} and OH radicals were produced via TME ozonolysis.

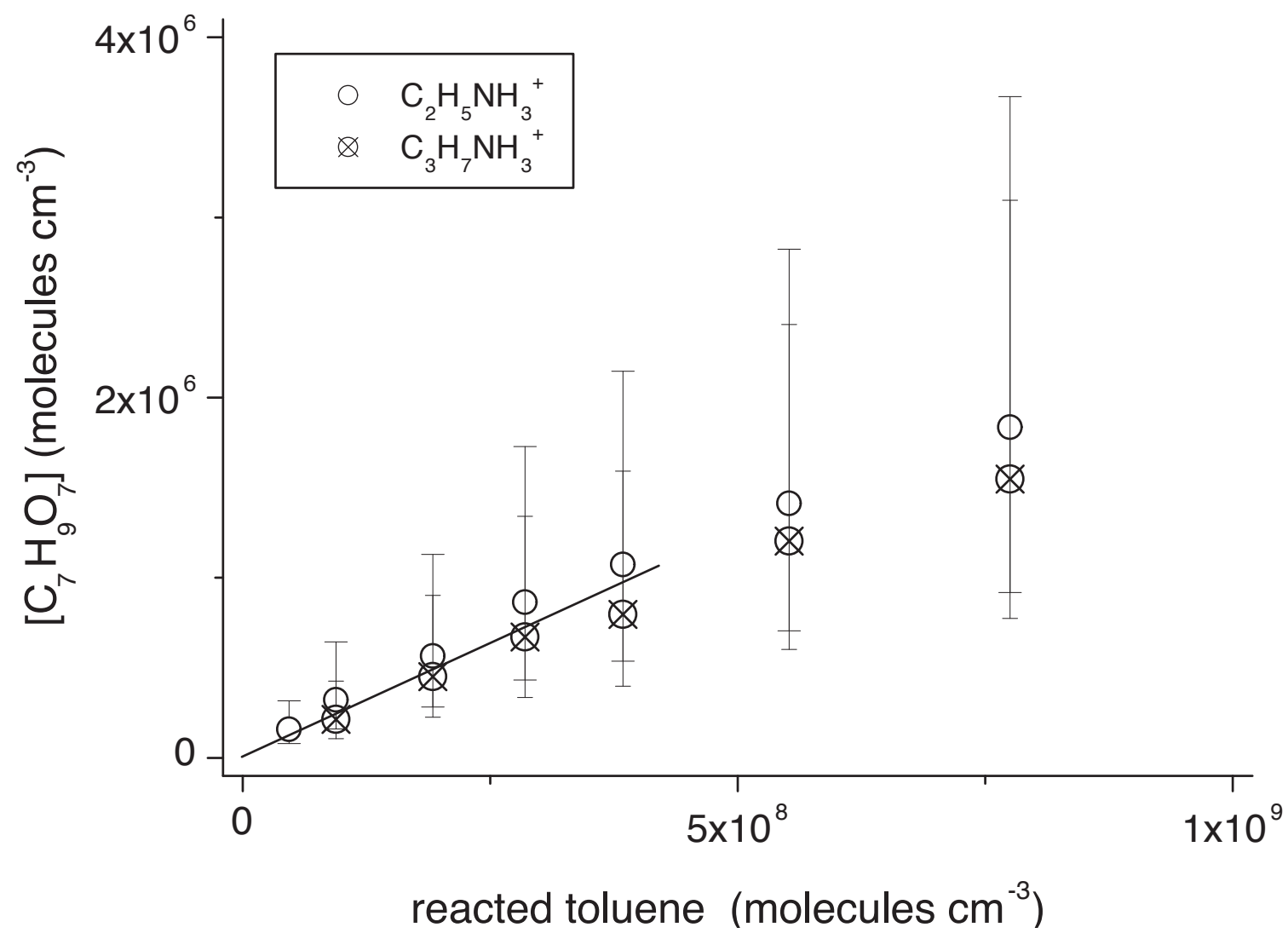

Supplementary Fig. 3 Concentration of $C_7H_9O_7$ as a function of reacted toluene. Product ionization was carried out by means of $C_2H_5NH_3^+$ and $C_3H_7NH_3^+$ ionization, see Fig. 2a. Only data for reacted toluene < 5×10^8 molecules cm^{-3} were considered for the slope because of a slight curvature for higher toluene conversion. The resulting molar $C_7H_9O_7$ formation yield is $0.25^{+0.25}_{-0.13}$ %.

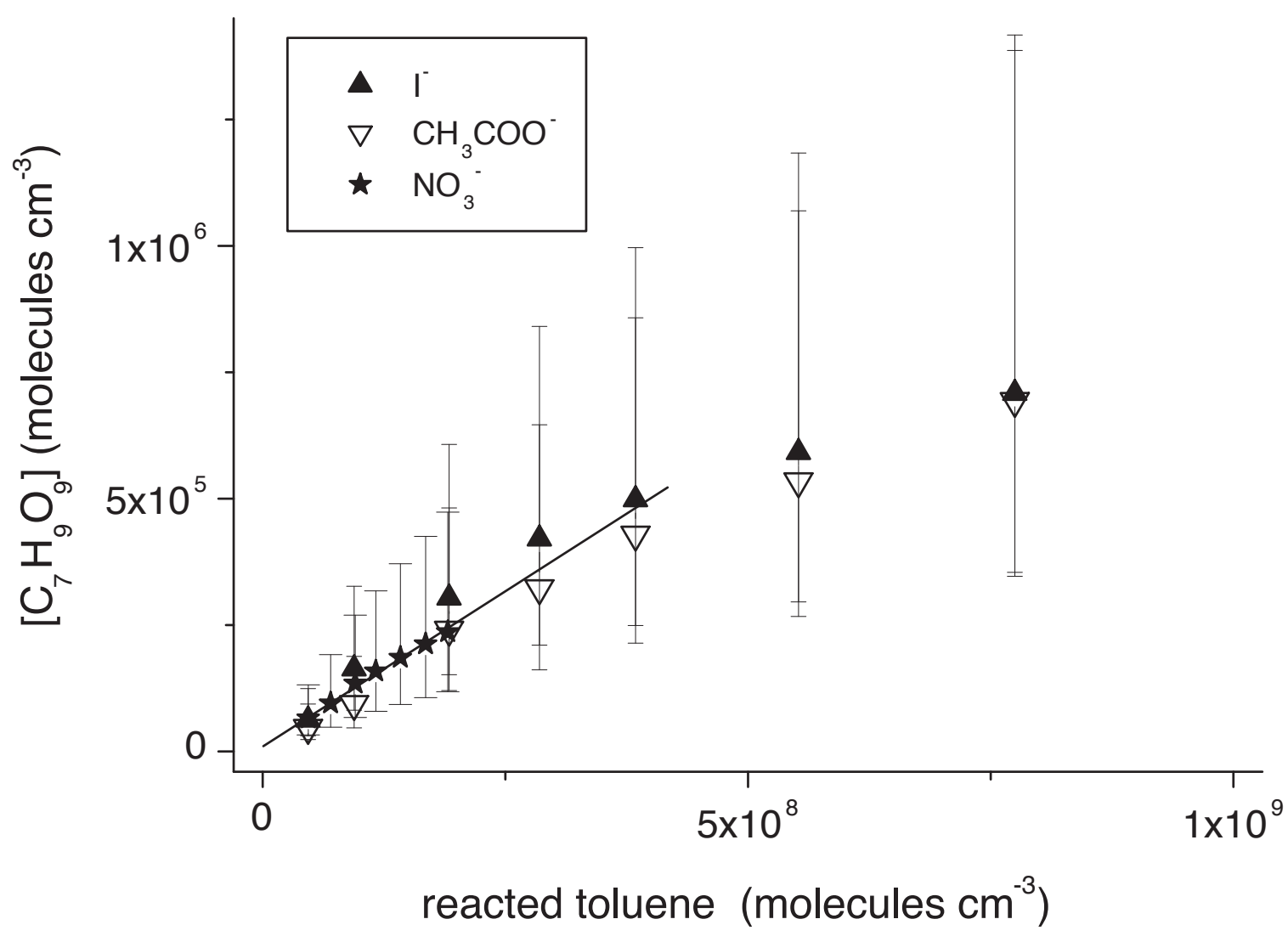

Supplementary Fig. 4 Concentration of $C_7H_9O_9$ as a function of reacted toluene. Product ionization was carried out by means of I^-, CH_3COO^- and NO_3^- ionization, see Fig. 2b. Only data for reacted toluene < 5×10^8 molecules cm^{-3} were considered for the slope because of a slight curvature for higher toluene conversion. The resulting molar $C_7H_9O_9$ formation yield is $0.12^{+0.12}_{-0.06}$ %.

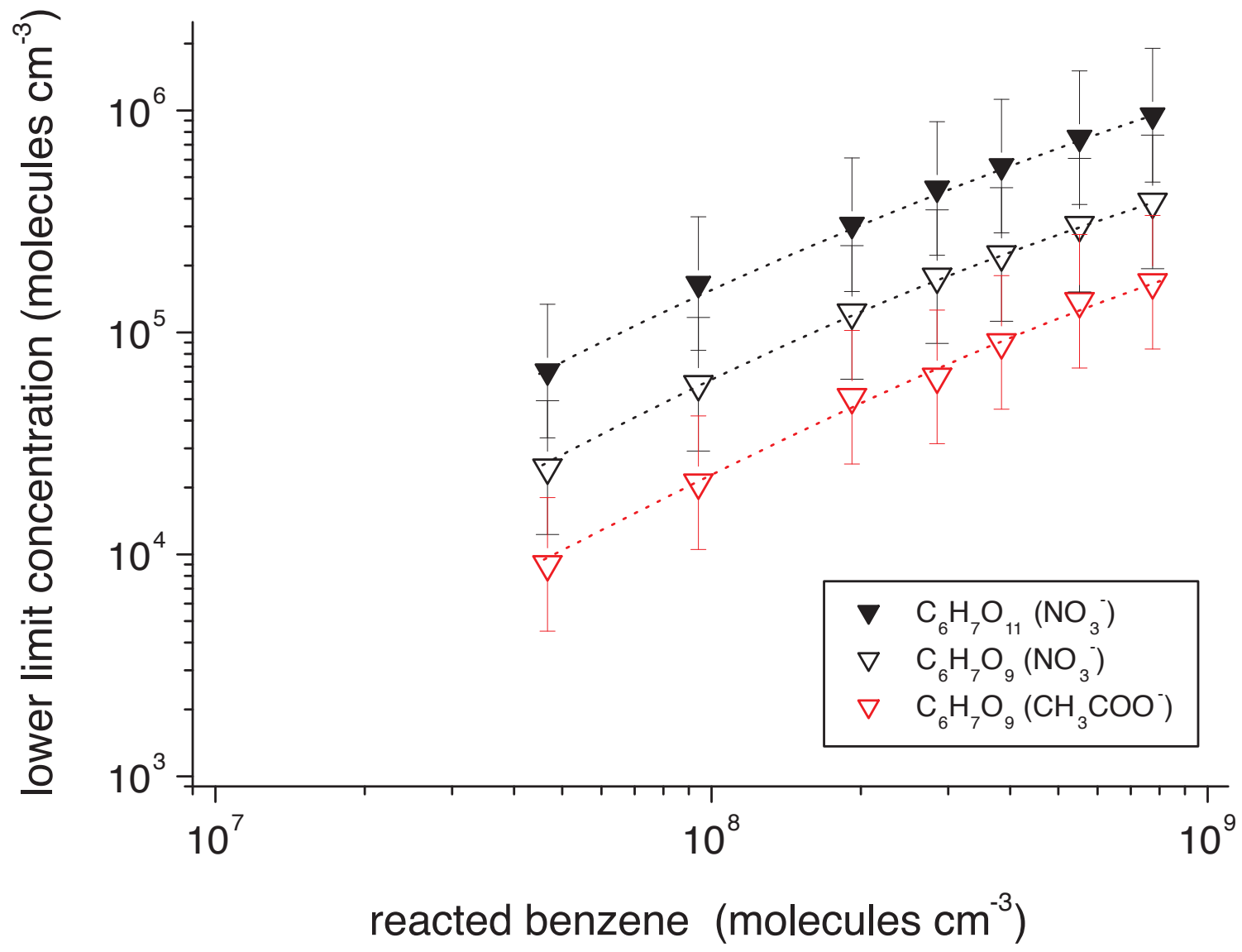

Supplementary Fig. 5 HOM-RO_2 radical generation from OH + C_6H_6 for low-NO conditions. OH radicals were produced via TME ozonolysis and CH_3COO^- and NO_3^- served as the reagent ions. The stated lower limit concentrations were obtained assuming collision limit in the ion-molecule reaction and negligible ion loss in the mass spectrometer (Methods). Error bares represent an uncertainty of a factor of two. The amount of reacted benzene was calculated by a model (Supplementary Note 1). Molar formation yields for a benzene conversion $< 4 \times 10^8$ molecules cm^{-3} are $0.15^{+0.15}_{-0.08}$ % ($C_6H_7O_{11}$) and $0.06^{+0.06}_{-0.03}$ % ($C_6H_7O_9$) based on the data obtained by NO_3^- ionization assuming maximum detection sensitivity. Reactant concentrations were (unit: molecules cm^{-3}): [TME] = 2.6×10^{10}, [O_3] = $(6.2 - 104) \times 10^{10}$ and [C_6H_6] = 2.1×10^{13}.

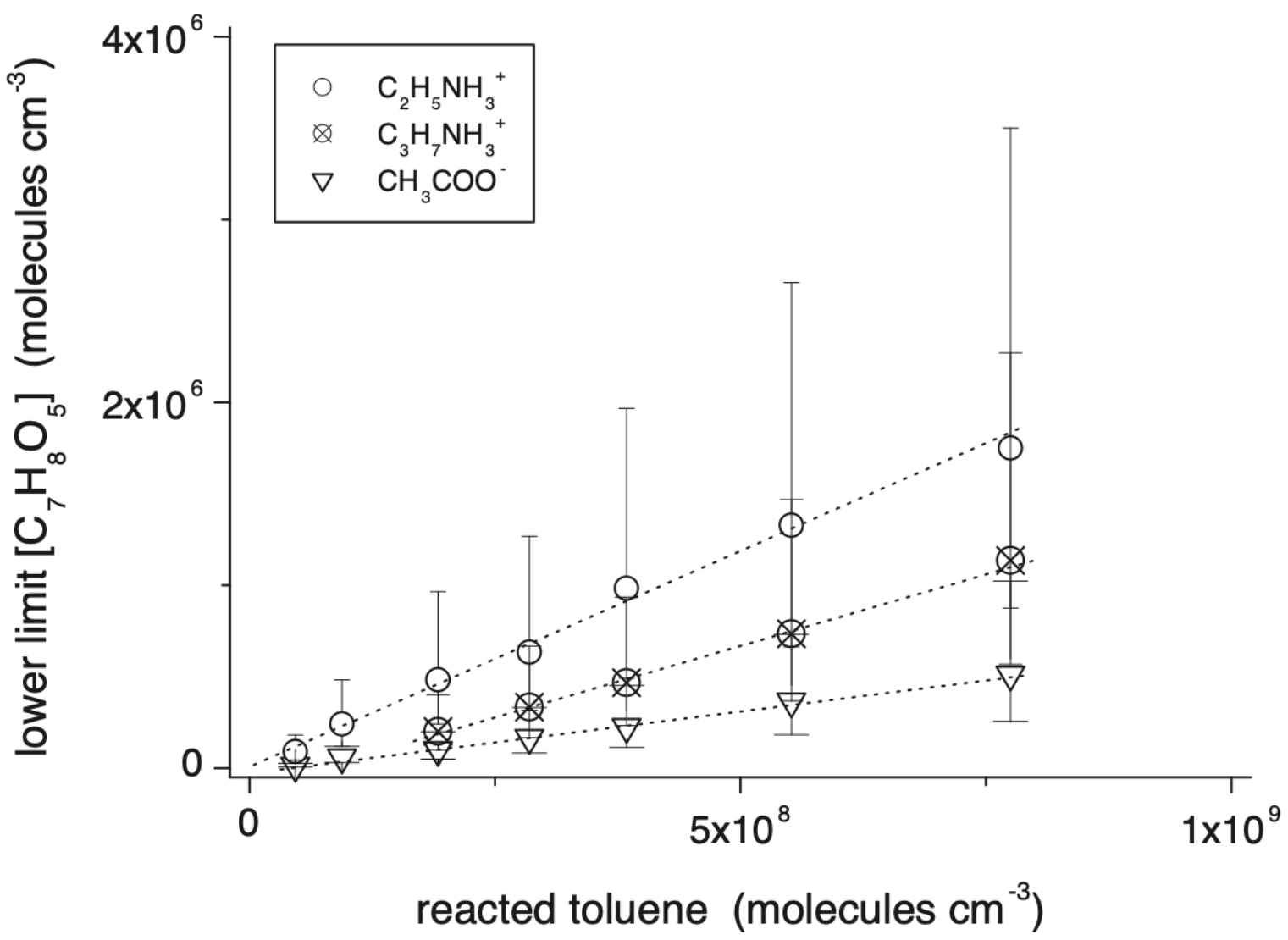

Supplementary Fig. 6 $C_7H_8O_5$ formation from OH + C_7H_8 for low-NO conditions measured by means of different ionization schemes. Concentrations were obtained applying the lower limit approach. Error bares represent an uncertainty of a factor of two. Product analysis was carried out from the experiments mentioned in Fig. 2. The $C_7H_8O_5$ molar formation yield is $0.24^{+0.24}_{-0.12}$ % resulting from measurements by means of $C_2H_5NH_3^+$ ionization. Close to maximum detection sensitivity is assumed for this ionization scheme.

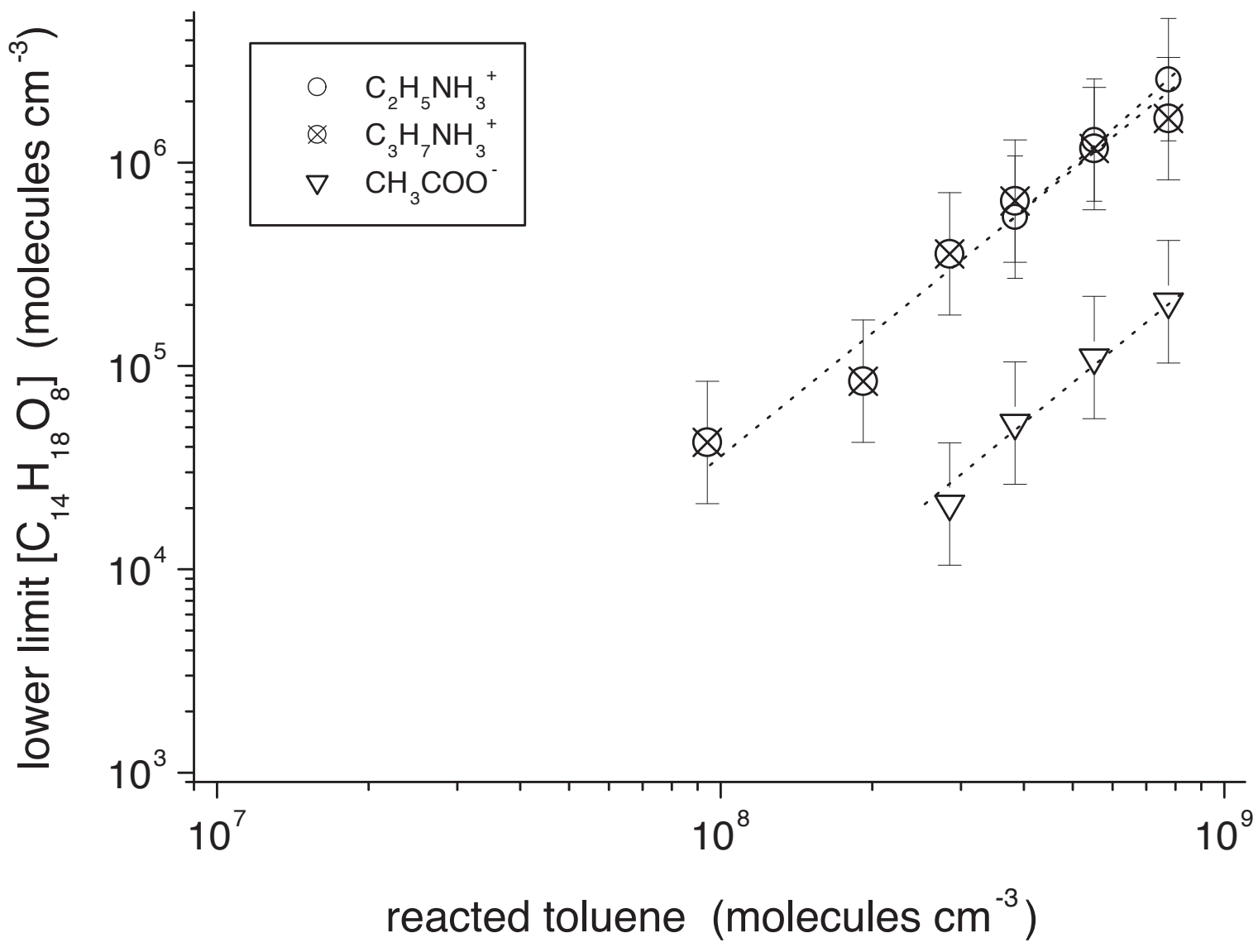

Supplementary Fig. 7 $C_{14}H_{18}O_8$ formation from OH + C_7H_8 for low-NO conditions. Stated concentrations represent lower limit values assuming collision limit in the ion-molecule reaction and negligible ion loss in the instrument (Methods). The error bares represent an uncertainty of a factor of two. Analysis was carried out from the experiments mentioned in Fig. 2.

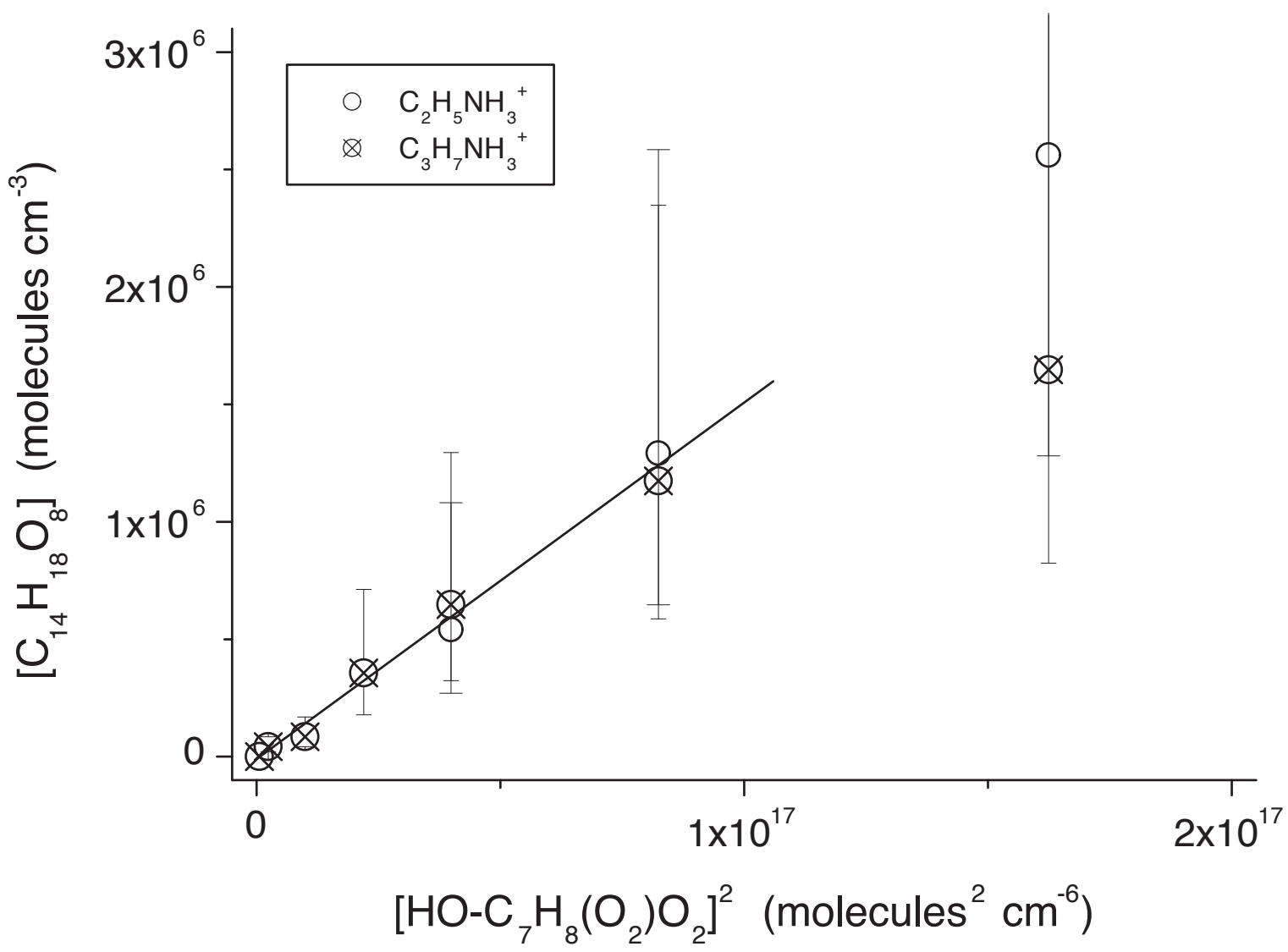

Supplementary Fig. 8 Kinetic analysis of $C_{14}H_{18}O_8$ formation. Needed concentrations were taken from the experiments given in Supplementary Fig. 7. Error bares represent an uncertainty of a factor of two in the measured concentrations. BCP (HO-$C_7H_8(O_2)O_2$) concentrations were calculated from reacted toluene considering the experimentally observed molar BCP yield of 52 %. The rate coefficient $k_1 = 1.4 \times 10^{-12}$ cm^3 $molecule^{-1}$ s^{-1} was obtained from the observed slope, [$C_{14}H_{18}O_8$] vs. [HO-$C_7H_8(O_2)O_2$]^2, of 1.5×10^{-11} cm^3 $molecule^{-1}$ based on equation (8).

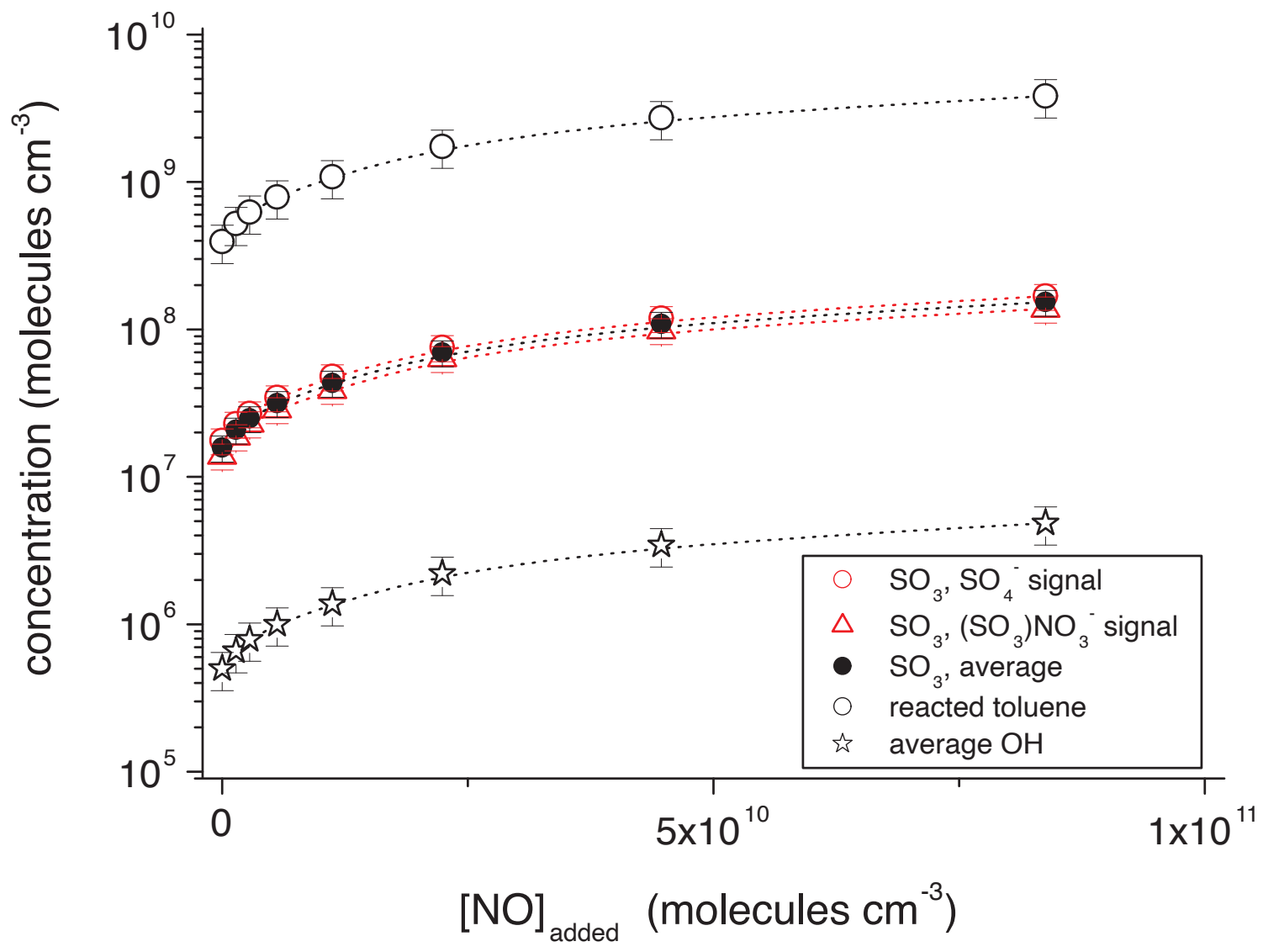

Supplementary Fig. 9 SO_3 formation from OH + C_7H_8 in the presence of SO_2 and the resulting amount of reacted toluene and average OH radical concentrations. SO_3 detection was carried out by means of nitrate ionization monitoring the signals of SO_4^- and $(SO_3)NO_3^-$. The uncertainty of SO_3 calibration is estimated to be not higher than ± 20 % as given by the error bars. Reacted toluene was calculated from the mean value of the resulting SO_3 concentrations determined from both measured signals considering the OH reactivity ($k_{OH+C7H8}$ × $[C_7H_8]$) / (k_{OH+SO2} × $[SO_2]$) = 25 according to the chosen reactant concentrations. The resulting uncertainty (Gauß´s propagation of uncertainty) of reacted toluene is estimated to be ± 30 %. Average OH radical concentrations with an uncertainty of ± 30 % follow from reacted toluene according $[OH]_{average} = \Delta[C_7H_8] / (k_{OH+C7H8} \times [C_7H_8] \times t)$. Reactant concentrations were (unit: molecules cm^{-3}): [IPN] = 7.7 × 10^{10}, $[C_7H_8]$ = 4.4 × 10^{12}, added [NO] = (1.4 - 84) × 10^9 and $[SO_2]$ = 1.13 × 10^{12}.

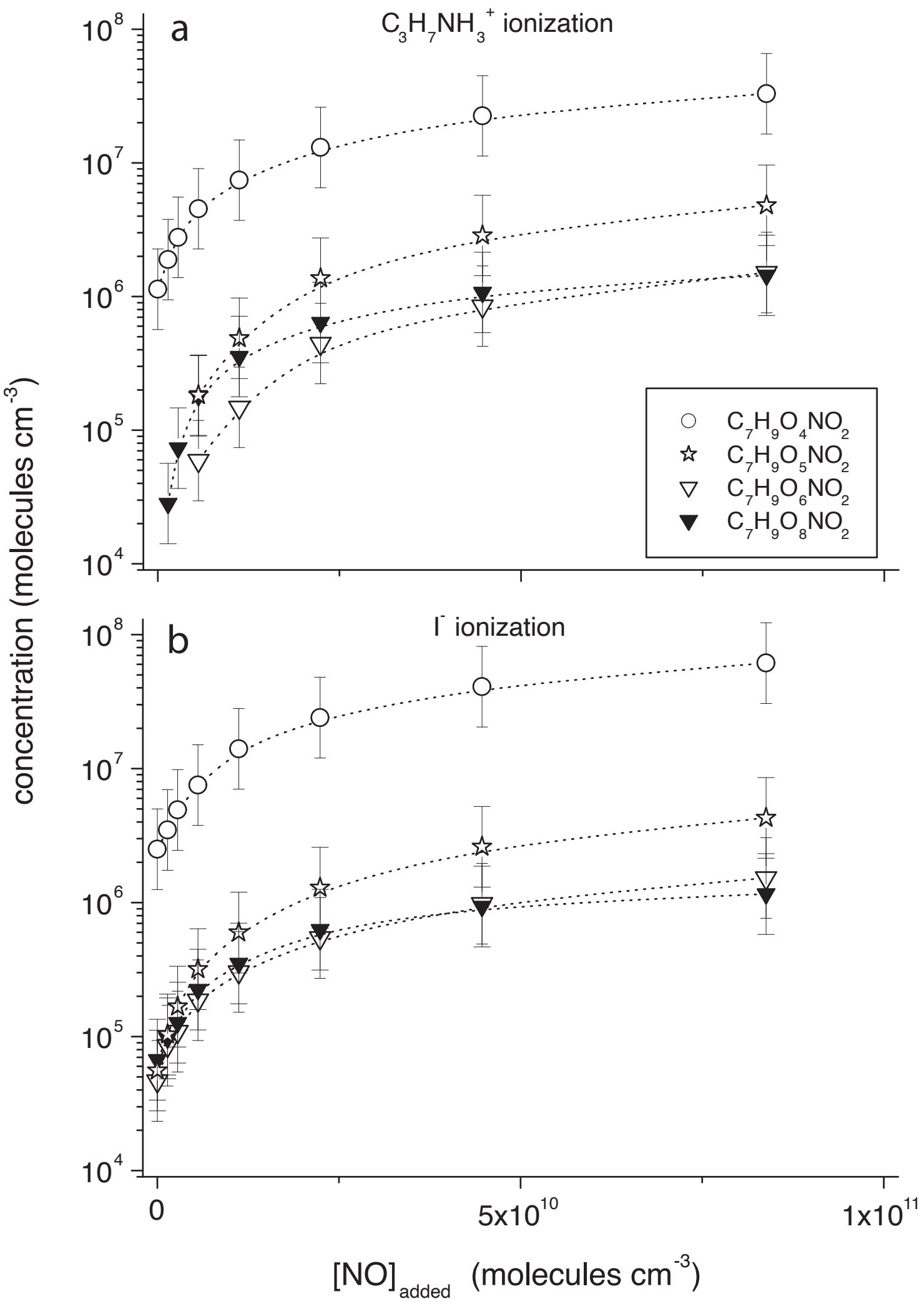

Supplementary Fig. 10 Measured concentrations of the organic nitrates $C_7H_9O_{4,5,6,8}NO_2$. Ionization was carried out by **a)** n-propylaminium $C_3H_7NH_3^+$ or **b)** iodide I^-. Concentrations were obtained applying the lower limit approach. Error bars show the uncertainty of a factor of two. The good agreement of the results from both ionization schemes within the range of uncertainty allow to assume that these concentrations are close to the "real" values. Reactant concentrations were (unit: molecules cm^{-3}): [IPN] = 7.7×10^{10}, [C_7H_8] = 4.4×10^{12} and added [NO] = $(1.4 - 84) \times 10^9$, see also Fig. 4.

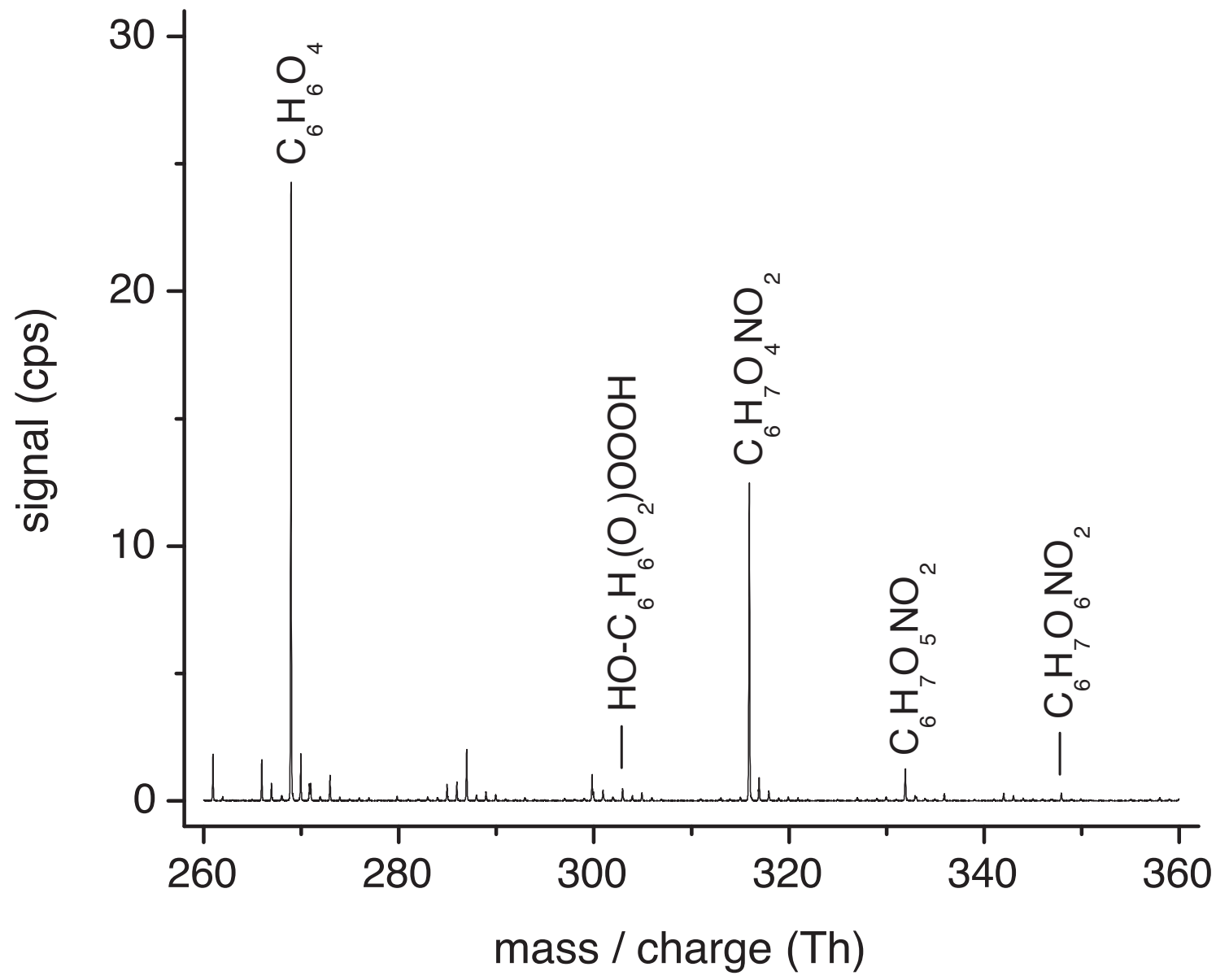

Supplementary Fig. 11 Mass spectrum from OH + C_6H_6 in the presence of NO applying I^- ionization. Product signals appear as adduct with the reagent ion. The selected range of 260 - 360 Th depicts detected C_6 products with at least four O atoms. Reactant concentrations were (unit: molecules cm^{-3}): [IPN] = 7.8×10^{10}, [C_6H_6] = 2.1×10^{13} and added [NO] = 7.1×10^{10} with a benzene conversion of 1.04×10^{10} molecules cm^{-3}.

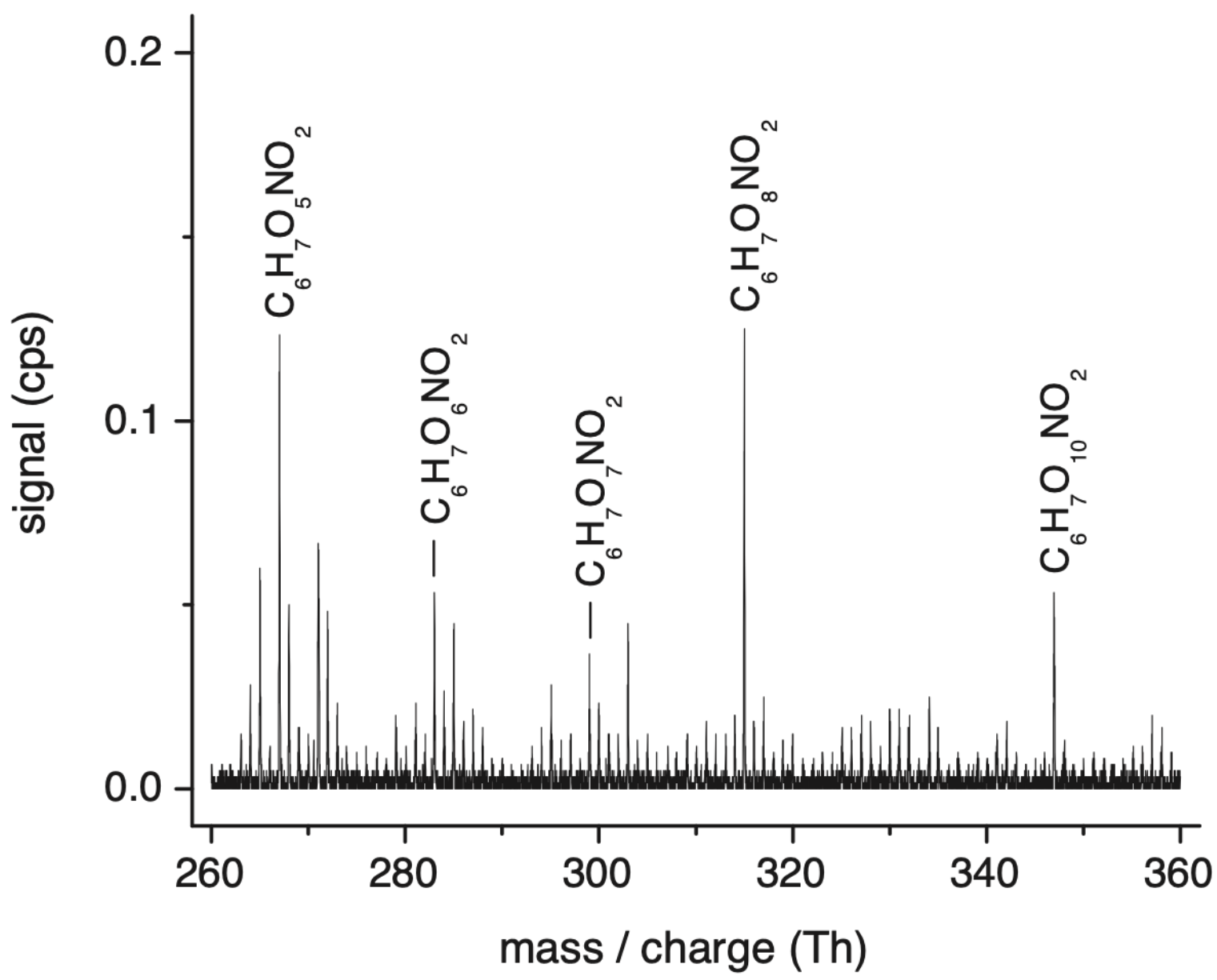

Supplementary Fig. 12 Mass spectrum from OH + C_6H_6 in the presence of NO using NO_3^- ionization. Product signals appear as adduct with the reagent ion. The experimental conditions are identical with those used for recording the I^- spectrum given in Supplementary Fig. 10.

Supplementary Note 1. Reaction scheme of OH + toluene/benzene describing the processes in the LFT for low-NO experiments using TME ozonolysis for OH radical formation. Rate coefficients at 295 K were taken from literature or from the present work or were estimated.

Reaction	Rate coefficient (cm^3 $molecule^{-1}$ s^{-1} or s^{-1})
O_3 + TME → 0.40×sCI + 0.52×OH + 0.08×HO_2 + 0.52×$CH_3C(O)CH_2O_2$	1.0×10^{-15} [1]
sCI → OH + $CH_3C(O)CH_2O_2$	900 [2]
OH + TME → products	1.1×10^{-10} [3]
OH + HO_2 → H_2O + O_2	1.1×10^{-10} [3]
HO_2 + HO_2 → H_2O_2 + O_2	1.65×10^{-12} [3]
O_3 + HO_2 → OH + 2 × O_2	2.0×10^{-15} [3]
OH + toluene → 0.52×BCP + 0.18×HO_2 + products	5.6×10^{-12} [4]
OH + benzene → 0.47×BCP + 0.53×HO_2 + products	1.2×10^{-12} [4]
BCP + $CH_3C(O)CH_2O_2$ → products	1.0×10^{-12} [estimated]
BCP + BCP → products	1.4×10^{-12} [this work]
BCP + NO → products	8.8×10^{-12} [5]
BCP + HO_2 → products	2.0×10^{-11} [estimated]

Supplementary references

1 Witter, M., Berndt, T., Böge, O., Stratmann, F. & Heintzenberg, J. Gas-phase ozonolysis: Rate coefficients for a series of terpenes and rate coefficients and OH yields for 2-methyl-2-butene and 2,3-dimethyl-2-butene. *Int J Chem Kinet* **34**, 394-403 (2002). https://doi.org/10.1002/kin.10063

2 Peltola, J., Seal, P., Vuorio, N., Heinonen, P. & Eskola, A. Solving the discrepancy between the direct and relative-rate determinations of unimolecular reaction kinetics of dimethyl-substituted Criegee intermediate $(CH_3)_2COO$ using a new photolytic precursor. *Phys Chem Chem Phys* **24**, 5211-5219 (2022). https://doi.org/10.1039/d1cp02270a

3 Atkinson, R. *et al.* Evaluated Kinetic and Photochemical Data for Atmospheric Chemistry: Supplement IV. IUPAC Subcommittee on Gas Kinetic Data Evaluation for Atmospheric Chemistry. *J Phys Chem Ref Data* **21** (1992). https://doi.org/10.1063/1.555918

4 Mellouki, A. *et al.* Evaluated kinetic and photochemical data for atmospheric chemistry: volume VIII – gas-phase reactions of organic species with four, or more, carbon atoms ($\geq$ C4). *Atmos Chem Phys* **21**, 4797-4808 (2021). https://doi.org/10.5194/acp-21-4797-2021

5 Wennberg, P. O. *et al.* Gas-Phase Reactions of Isoprene and Its Major Oxidation Products. *Chem Rev* **118**, 3337-3390 (2018). https://doi.org/10.1021/acs.chemrev.7b00439
